# SBRF-Det: Scale-Boundary Representative Fusion for Multi-Finding Detection in Panoramic Dental Radiographs

**DOI:** 10.3390/diagnostics16193259

**Published:** 2026-10-08

**Authors:** Furkan Karatas, Omer Aslan, Mehmet Burukanli, Shahid Mohammad Ganie, Merve Ersoy, Faruk Ozger, Ishak Pacal

**Affiliations:** 1 Department of Prosthetic Dentistry, Faculty of Dentistry, Igdir University, Igdir 76000, Turkey; furkan.karatas@igdir.edu.tr; 2 Department of Prosthetic Dentistry, Faculty of Dentistry, Ataturk University, Erzurum 25240, Turkey; omer.aslan21@ogr.atauni.edu.tr; 3 Department of Computer Engineering, Faculty of Engineering and Architecture, Bitlis Eren University, Bitlis 13000, Turkey; mburukanli@beu.edu.tr; 4 Department of Health Information Management and Technology, College of Applied Medical Sciences, King Faisal University, Al-Ahsa 31982, Saudi Arabia; 5 Department of Software Engineering, Faculty of Engineering and Natural Sciences, Istanbul Topkapi University, Istanbul 34000, Turkey; merveersoy@topkapi.edu.tr; 6 Department of Computer Engineering, Faculty of Engineering, Igdir University, Igdir 76000, Turkey; 7 Department of Electronics and Information Technologies, Faculty of Architecture and Engineering, Nakhchivan State University, Nakhchivan AZ 7012, Azerbaijan; 8 Department of Computer Engineering, Faculty of Engineering and Natural Sciences, Fenerbahce University, Istanbul 34758, Turkey

**Keywords:** panoramic dental radiography, artificial intelligence, computer-aided detection, multi-finding detection, representative modeling, boundary-aware feature fusion

## Abstract

**Background**: Panoramic dental radiographs frequently contain multiple coexisting pathological, anatomical, and treatment-related findings that vary in scale, contrast, and boundary definition. Although recent one-stage detectors provide strong performance, reliable localization of small or weakly defined findings remains challenging. **Methods**: We propose SBRF-Det, a one-stage multi-finding detector integrating scale-dependent representative allocation with boundary-conditioned local–global feature fusion at P4/16. The method was evaluated on OralXrays-9, comprising 12,688 panoramic radiographs and 84,099 retained annotations across nine categories, using a fixed 2688-image hold-out cohort. Twenty-two detector configurations were compared under a common protocol, and A0–A2 were further assessed using three independent seeds and paired radiograph-level bootstrap analysis. **Results**: SBRF-Det achieved 0.8777 precision, 0.8979 recall, 0.9152 mAP50, and 0.7029 mAP50-95. Relative to the matched YOLO12l baseline, mAP50-95 increased by 0.95 percentage points. Performance remained class dependent, with apical periodontitis reaching 0.4526 mAP50-95 versus 0.8401–0.8842 for implants, porcelain crowns, and ceramic bridges. SBRF-Det used 93.06 GFLOPs versus 95.99 for A0, but inference latency increased from 5.946 to 8.851 ms/image. Three-seed experiments preserved the A0–A1–A2 ordering, while paired bootstrap intervals for A2–A0 and A2–A1 excluded zero. **Conclusions**: SBRF-Det improves strict localization through representative and boundary-aware feature modeling, but the gain is modest and introduces a runtime trade-off. Patient-linked, multi-center, and reader-centered validation remains necessary before clinical deployment.

## 1. Introduction

Oral diseases remain among the most common chronic health conditions and affect patients across virtually all age groups. Dental caries, periodontal disease, tooth loss, periapical pathology, developmental abnormalities, and treatment-related conditions may progressively impair mastication, aesthetics, and quality of life when diagnosis or intervention is delayed [1]. Periodontitis is particularly important because destruction of the supporting tissues may continue with limited symptoms until substantial bone loss and tooth mobility have developed [2]. Dental caries follows a similarly progressive course and, when untreated, may lead to pain, pulpal involvement, infection, and eventual tooth loss, making timely recognition a central part of preventive and minimally invasive care [3]. In routine dental practice, several pathological, developmental, and treatment-related conditions may coexist within the same examination, making panoramic interpretation inherently multi-finding rather than a sequence of isolated binary decisions.

Radiographic examination provides information that cannot be obtained reliably from visual inspection alone, particularly for roots, alveolar bone, impacted teeth, periapical regions, and previously treated structures. Panoramic radiography is widely used because one acquisition covers the maxilla, mandible, dentition, temporomandibular joints, and adjacent anatomical structures, providing a broad view for diagnosis and treatment planning [4]. The same wide field of view complicates interpretation because three-dimensional anatomy is projected onto a two-dimensional plane, while superimposition, geometric distortion, local image-quality variation, and overlapping structures can obscure subtle abnormalities. Dental findings also vary markedly in size, shape, radiographic density, and anatomical position, and several targets may occur in close spatial proximity. Manual assessment can therefore be time-consuming and observer dependent, especially when the entire image must be screened for multiple findings rather than a single predefined target [5]. A clinically useful computer-aided detector should consequently localize coexisting findings and preserve the spatial evidence required for clinician verification.

Deep learning has provided a practical route toward automated dental-image analysis by learning task-relevant representations directly from annotated images. Recent panoramic applications include caries, periapical lesions, periodontal assessment, cysts and tumors, tooth segmentation, restorations, and other diagnostic targets [6]; within object detection, successive YOLO generations have supported efficient tooth- and finding-level localization across a wide range of model capacities [7]. Their performance, however, remains task dependent: later detector versions do not consistently improve every dental condition, as illustrated by category-specific differences in traumatic injuries and by a 13-condition comparison in which YOLOv11 outperformed YOLOv12 on internal and external data [8,9]. Digital dentistry has also expanded into material processing, restorative color control, and data-driven aesthetic support, including studies of zirconia processing, resin-cement color matching, and machine-learning-based tooth-shade assessment under variable clinical illumination [10,11,12,13]. Beyond dentistry, explainable brain-tumor MRI classification, breast-ultrasound classification, ischemic-stroke segmentation, and real-time colorectal polyp detection demonstrate the broader ability of deep models to handle heterogeneous medical-imaging tasks in which local detail and wider anatomical context must be balanced [14,15,16,17].

The remaining challenge is therefore not simply to select the newest or largest detector. A systematic review of 73 YOLO-based dental imaging studies identified substantial variation in datasets and evaluation practice, including frequent reliance on small or single-center cohorts, augmentation-heavy training, limited external validation, and incomplete reporting of stricter localization metrics such as mAP50-95 [18]. OralXrays-9 provides a more demanding setting, with 12,688 panoramic radiographs and 84,113 annotated instances across nine categories that differ substantially in scale, morphology, anatomical location, and frequency [19]. Related multi-finding work has likewise shown that small lesions, low-contrast regions, and adjacent abnormalities require deliberate multi-scale feature aggregation and context modeling [20]. The resulting gap is architectural: a detector for panoramic multi-finding analysis must preserve weak local evidence while maintaining sufficient contextual information for large structures and spatially separated abnormalities.

To address this gap, this study develops RP-P4 as a detector-specific representative feature pathway at the intermediate P4/16 stage and extends it to SBRF-Det through a scale-dependent representative-count rule and boundary-conditioned local–global fusion. The design targets compact long-range communication without dense pairwise spatial attention while retaining fine structural evidence relevant to strict localization. We hypothesized that representative communication at the intermediate P4/16 stage, when combined with boundary-conditioned local structural processing, would improve strict multi-finding localization relative to a matched conventional P4/16 baseline without requiring a substantial increase in model size. The scale-dependent representative-count rule was implemented as part of the architecture, but its isolated effect was not treated as a separately validated causal contribution. Evaluation uses a fixed development/hold-out protocol and a broad benchmark of contemporary one-stage detectors under matched data partitions, a common framework-level training policy, image resolution, and hardware. The study further examines multi-seed robustness, paired uncertainty in incremental gains, class-specific behavior, calibration, and computational trade-offs so that the proposed architecture is assessed as a complete detection system rather than by a single headline score. The main contributions are as follows:

- RP-P4 is introduced as a detector-specific representative feature pathway at P4/16, providing compact long-range contextual communication while retaining the surrounding multi-scale neck and detection head.
- SBRF-Det extends RP-P4 with a scale-dependent representative-count rule and boundary-conditioned fusion of global representative context with local structural evidence. The integrated formulation is evaluated directly, while the isolated benefit of adaptive representative count is reserved for dedicated sensitivity analysis.
- A controlled A0–A1–A2 analysis separates the matched baseline, RP-P4, and the complete SBRF-Det design, with mAP50-95 increasing progressively from 0.6934 to 0.7009 and 0.7029.
- The released 2688-image evaluation cohort is retained as an untouched hold-out test set, while development splitting and exact-content duplicate controls are documented explicitly; the absence of distributed patient identifiers is treated as a limitation rather than inferred away.
- Twenty-two detector configurations are compared under a common benchmark protocol using strict localization, class-specific performance, parameter count, GFLOPs, batch-1 latency, bootstrap confidence intervals, and calibration, providing an accuracy-efficiency and reliability-oriented evaluation relevant to computer-aided panoramic finding detection.
- A controlled component-removal study independently tests the representative, local-structural, and boundary-conditioned pathways, and a uniform batch-1 re-profiling protocol quantifies parameters, GFLOPs, inference and pipeline latency, throughput, and peak GPU memory for the focal ablation variants.

## 2. Related Work

### 2.1. Deep Learning for Specific Findings in Panoramic Radiographs

Early dental deep-learning studies usually treated panoramic interpretation as a narrowly defined detection or segmentation problem. This task-centered design made it possible to optimize models for a particular radiographic pattern, but it also limited what could be inferred about performance when several unrelated findings coexist in the same image. Ragab et al. introduced YOLOv7-M for periodontal bone-loss detection by adding a focus module and feature-fusion mechanism, reporting an F1-score of 92.5% and an mAP of 91.0% [21]. Sarı et al. compared YOLOv8, YOLOv9, and Faster R-CNN for dens invaginatus in 400 panoramic radiographs; YOLOv9 achieved the highest F1-score of 0.937, whereas YOLOv8 provided the shortest inference time [22]. Kaygısız et al. applied YOLOv11-S to dentigerous, odontogenic keratocyst, and radicular cyst detection and obtained an mAP of 86% and an F1-score of 83% on 311 panoramic images [23]. Ayhan et al. examined caries beneath fixed dental prostheses using YOLOv7 and YOLOv7+CBAM, with the attention-enhanced variant increasing mAP from 0.800 to 0.846 [24].

Other work has extended object detection toward structured dental assessment rather than simple lesion localization. Le et al. combined YOLOv11-based tooth segmentation with anatomical landmark detection to automate radiographic bone-loss measurement and periodontitis staging; the segmentation stage reached an mAP50 of 0.994, although the workflow remained organized around periodontal analysis [2]. Uzel et al. evaluated YOLOv8 for supernumerary teeth in pediatric panoramic radiographs and reported perfect classification on their evaluated sets, but the segmentation stage showed 38% recall and an F1-score of 55%, revealing a clear difference between image-level recognition and reliable localization [25]. The pattern across these studies is consistent: strong results are achievable when the target is well constrained, yet performance and model behavior change substantially when localization is subtle or the target population is small. Such evidence does not establish how a detector will handle a panoramic image containing lesions, restorations, missing teeth, implants, and developmental findings at the same time.

### 2.2. Comparative Evaluation of Recent Detection Architectures

The rapid release of new detector generations has encouraged direct comparisons under common dental tasks. These studies are useful because they separate architectural effects from changes in the clinical target, and they also show that model chronology is a poor substitute for empirical evaluation. Şevik and Mutlu compared YOLOv8, YOLOv9, YOLOv10, and YOLOv11 for four pediatric dental findings; YOLOv11x reached an internal mAP50 of 0.91 and an external mAP50 of 0.82 [26]. Mutlu et al. later evaluated YOLOv11 and YOLOv12 across 13 dental conditions using 2297 panoramic radiographs and found YOLOv11 to be the stronger model, with mAP50 values of 0.857 internally and 0.806 externally [9]. Sarıoğlu et al. compared YOLOv8x, YOLOv11x, and YOLOv12x for traumatic dental injuries. YOLOv12 produced the highest overall multiclass F1-score, but periodontal injuries remained difficult for every model, with markedly lower performance than hard-tissue injuries [8].

Broader architectural comparisons lead to the same conclusion. Khurshid et al. evaluated YOLOv11, Faster R-CNN, and a Vision Transformer on 2235 panoramic radiographs from three dental colleges; the Vision Transformer produced the highest reported accuracy and F1-score, whereas Faster R-CNN achieved the highest mAP50 [5]. Yilmaz et al. compared Transformer-, CNN-, and YOLO-based models for mandibular condyle segmentation and found RT-DETR to have the highest numerical Dice score, while YOLOv9-Seg remained a competitive real-time alternative [27]. These results show that detector ranking changes with the task, metric, and structure being analyzed. More importantly, the failure modes reported for subtle lesions and low-contrast regions persist even when larger or newer networks are used, which argues for task-specific changes to feature extraction and fusion rather than routine replacement of one YOLO generation with the next.

### 2.3. Multi-Finding Detection and Task-Oriented Detector Architectures

Recent work has increasingly modified the detector itself to address the characteristics of panoramic dental images. The emphasis has shifted from selecting a standard YOLO variant toward changing how features are extracted, fused, and localized across scales. Hua et al. proposed YOLO-DentSeg by augmenting YOLOv8n-seg with a lightweight C2f-Faster block, bidirectional feature fusion, channel attention, and an IoU-based localization objective; the resulting model reached mAP50 values of 0.872 for detection and 0.855 for segmentation with 1.81 million parameters [1]. Zeng et al. developed MMC-YOLO for joint object detection, instance segmentation, and periodontal assessment using progressive multi-scale feature aggregation, coordinate-guided fusion, and edge-enhanced spatial fusion on 2467 panoramic radiographs [20]. Yeahea et al. evaluated the nano variants of YOLOv8, YOLOv11, and YOLOv12 for multi-class instance segmentation of implants, missing teeth, root pieces, and impacted teeth; YOLOv11n-seg achieved the best overall box and mask mAP50 while retaining low inference cost [28].

Large-scale benchmarks make the limitations of narrower studies more visible. Chen et al. introduced OralXrays-9 with 12,688 panoramic radiographs and 84,113 annotations across nine categories, and their Multi-Object Query-Aware Mining framework explicitly targeted scale variation and class imbalance [19]. In a different task, Annamalai and Parvathy used a YOLO-OBB-derived DenSE-Net for maxillary canine impaction and obtained mAP50 of 0.969 and mAP50-95 of 0.671, illustrating the value of adapting localization geometry to the structure being modeled [29]. Hartman et al., reviewing 73 YOLO-based dental imaging studies, nevertheless found persistent weaknesses in dataset size, external validation, and metric consistency [18]. The direction of the field is therefore clear, but the evidence is still concentrated in single-disease or small-class settings. A large nine-class benchmark provides a stricter test of whether an architectural modification can preserve small-object detail, accommodate large structural targets, and remain effective across severe differences in class frequency; this is the setting addressed in the present study.

To distinguish mechanism-level novelty from cross-study performance differences, Table 1 positions the proposed pathway against representative/global-context strategies that are conceptually relevant to the present design. The table is intended as an architectural comparison rather than a head-to-head accuracy ranking because the cited methods were developed for different tasks, datasets, and evaluation protocols.

The distinction is therefore not that representative interaction alone is new. The contribution lies in adapting compact representative communication to the detector’s intermediate P4/16 stage and coupling it with scale-dependent representative allocation, local structural processing, and boundary-conditioned fusion while leaving the multi-scale neck and prediction head unchanged.

## 3. Materials and Methods

### 3.1. Dataset, Diagnostic Targets, and Study Design

The experiments were conducted on OralXrays-9, a large panoramic radiography dataset developed for multi-object dental finding localization [19]. It contains 12,688 panoramic radiographs from 11,480 patients and bounding-box annotations for nine categories: apical periodontitis, decay, wisdom tooth, missing tooth, dental filling, root canal filling, implant, porcelain crown, and ceramic bridge. A radiograph may contain several categories and multiple instances of the same category; the task was therefore formulated as multi-class object detection rather than image-level classification.

The source annotations are bounding-box based and do not provide a unique tooth identifier. Consequently, the benchmark does not impose a mutually exclusive single-label assignment at tooth level: multiple class-specific boxes may occur within the same or overlapping tooth region when more than one finding is annotated. This is compatible with the YOLO formulation used here, in which candidate boxes are scored independently by class rather than forcing one diagnosis per radiograph or tooth region. Co-located and immediately adjacent findings were therefore evaluated at the box level and inspected during the dental review. A tooth-ID-based prevalence estimate of same-tooth coexistence was not calculated because the released metadata do not define tooth-instance identities.

The nine labels encompass active disease, tooth-status findings, and treatment-related structures rather than a single pathological entity. Apical periodontitis denotes inflammatory change in the periapical region, whereas decay represents carious tissue loss that may appear as a relatively subtle radiolucent defect [3,6]. Wisdom tooth and missing tooth encode eruption/anatomical status that can influence diagnosis and treatment planning, while dental filling and root-canal filling represent restorative and endodontic interventions [6,19]. Implant, porcelain crown, and ceramic bridge are prosthetic rehabilitation findings that are generally more radiopaque and structurally defined than early carious or periapical changes [5,19]. This mixture of low-contrast pathology and high-contrast restorative material produces large differences in object size, edge sharpness, texture, and anatomical context, which makes the benchmark a heterogeneous localization problem rather than a uniform lesion-detection task.

The dataset combines pronounced class imbalance with substantial variation in target size and radiographic appearance. Apical periodontitis and decay frequently occupy small, low-contrast regions, whereas teeth, implants, crowns, and bridges tend to form larger and more sharply defined structures. The source release contains 84,113 bounding-box annotations. During pre-model quality control, automated validity checks and radiographic overlay inspection were followed by joint review of flagged cases by two dental investigators. Fourteen annotations showed an inconsistency between the recorded box/label and the visible radiographic finding and were excluded before training. No radiograph was removed solely because of these annotation exclusions, and no excluded annotation was relabeled or replaced. The resulting analysis corpus therefore contained 84,099 retained annotations. The distributed dataset materials available for this analysis did not provide sufficient annotator-level information to reconstruct the original labeling workflow, individual annotator qualifications, adjudication sequence, or interobserver-agreement statistics. These quantities are therefore not inferred. The two-dentist quality-control procedure described here pertains to the retained analysis corpus and the interpretation of model outputs, and should not be interpreted as a retrospective estimate of agreement among the original dataset annotators. Wisdom tooth was the most frequent category with 27,325 instances, while missing tooth and implant contained 4092 and 4529 instances, respectively. The natural class distribution was preserved in all data partitions.

The released OralXrays-9 data comprise a 10,000-image development cohort and a separate 2688-image evaluation cohort. The released evaluation cohort was preserved unchanged as the final hold-out test set. Before development splitting, cryptographic file hashes were calculated for every image in both released cohorts and compared across cohorts; no byte-identical image occurred in both the 10,000-image development cohort and the 2688-image hold-out cohort. Exact-content duplicate groups within the development cohort were kept intact during splitting, and the 2688 hold-out images were mutually unique by the same exact-content screening. The development cohort was then divided into 8000 training and 2000 validation radiographs using multi-label stratification with a fixed random seed of 42. Because explicit patient identifiers were not available in the distributed metadata, these exact-content checks cannot establish patient-level independence or exclude non-identical repeated examinations from the same individual; patient-level regrouping was therefore not inferred from file names or other indirect identifiers. The resulting image and annotation distribution is summarized in Table 2.

### 3.2. Clinical Definition and Panoramic Radiographic Appearance of the Target Findings

The nine detector labels represent distinct clinical entities rather than interchangeable image classes. To ensure clinically appropriate interpretation of the benchmark categories, the dental co-authors reviewed the class definitions in relation to established endodontic, restorative, radiographic, and prosthodontic references. Table 3 summarizes both the clinical meaning of each target and the panoramic appearance that informed qualitative interpretation of the model outputs. These descriptions were used to contextualize the detection task and the subsequent dentist-reviewed error analysis; they were not used to alter the source annotations or to introduce additional labels.

The clinical heterogeneity in Table 3 also explains why aggregate detector performance must be interpreted cautiously. Radiopaque prosthetic structures such as implants, crowns, and bridges generally offer sharper boundaries and stronger contrast, whereas caries and periapical inflammatory changes may be smaller, less conspicuous, or partially superimposed by adjacent anatomy. Accordingly, class-specific localization metrics and failure patterns were evaluated alongside the overall benchmark rather than treating all nine labels as radiographically equivalent.

**Figure 1:**
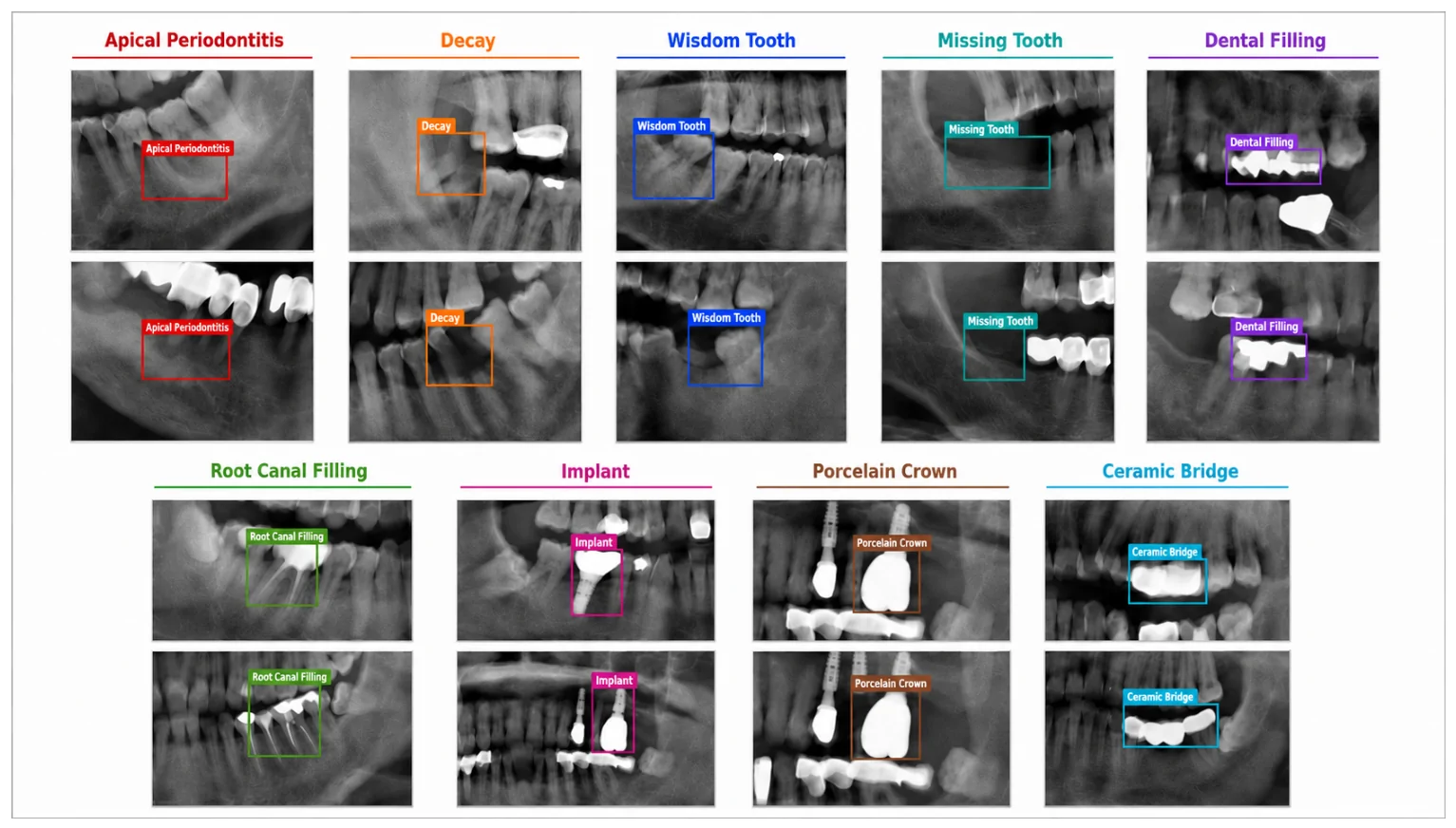
Representative panoramic radiograph regions from the nine OralXrays-9 categories. The examples illustrate the marked differences in object scale, shape, contrast, and anatomical context encountered by the detector.

The hold-out cohort exhibits a different category distribution from the development data, particularly for implant, root canal filling, porcelain crown, and ceramic bridge. No attempt was made to rebalance this cohort because altering its composition would change the predefined evaluation population. Representative examples of all nine categories are shown in Figure 1.

### 3.3. Annotation Processing and Data Partitioning

All annotation processing was completed before model training. Bounding boxes were checked for valid dimensions and consistency with image boundaries. Invalid boxes were removed, and coordinates extending beyond the image extent were constrained to valid limits. For an image of width W and height H, a box defined by upper-left coordinates (x, y), width w, and height h was converted to normalized center coordinates as defined in Equation (1).

(1) $$x_{c}=\frac{x+w/2}{W},\;y_{c}=\frac{y+h/2}{H}$$

The corresponding normalized box width and height were calculated according to Equation (2). Together, Equations (1) and (2) express every annotation relative to the radiograph dimensions, providing a common coordinate system across images with different native sizes. Because only the box parameterization is changed, the transformation does not alter the underlying lesion geometry or its association with the source image.

(2) $$w_{n}=\frac{w}{W},\;h_{n}=\frac{h}{H}$$

In Equations (1) and (2), the first pair defines the normalized box center and the second pair defines the normalized box width and height. The conversion changes only the coordinate representation and leaves the spatial correspondence between each annotation and its radiograph unchanged.

The public release does not provide explicit patient identifiers that permit reliable reconstruction of the patient-to-image relationship. Patient-level regrouping was therefore not inferred from file names or other indirect identifiers. The predefined 2688-image evaluation cohort was preserved unchanged for final testing, while the 10,000-image development cohort was separated into 8000 training and 2000 validation images using multi-label stratification. Exact-content duplicate screening was applied to the development cohort, and byte-identical images were assigned to the same development subset.

The three partitions had distinct roles throughout the experiments. Training images were used for parameter optimization, validation images for convergence monitoring and checkpoint selection, and the hold-out cohort exclusively for final evaluation. The test set was excluded from architecture selection, augmentation decisions, hyperparameter adjustment, and checkpoint selection. Exact-content duplicate screening of the hold-out cohort identified 2688 unique images and no duplicates.

Exact-content screening was complemented by two non-identical-image similarity analyses. A 64-bit perceptual hash was computed for every development and hold-out radiograph, and global image embeddings were extracted with an ImageNet-pretrained ResNet-50. No exact perceptual duplicate was detected across the development–hold-out boundary; the minimum cross-partition pHash Hamming distance was 15. The embedding analysis nevertheless identified highly similar radiographs, with a maximum development-to-hold-out cosine similarity of 0.9998009. Because high visual similarity does not establish patient identity, these pairs were treated as potential repeated or closely similar examinations rather than as verified same-patient duplicates. In a secondary sensitivity analysis, progressively excluding the most embedding-similar hold-out examinations preserved the A2–A0 mAP50-95 advantage at approximately +0.92 to +0.96 percentage points in the secondary evaluator. These checks reduce concern about direct image duplication but cannot establish patient-level independence in the absence of stable subject identifiers.

Annotation integrity was verified by checking class identifiers, finite coordinates, positive box dimensions, and image-bound consistency. Bounding boxes were projected back onto 150 randomly sampled radiographs, 50 from each partition, to identify possible coordinate displacement or label inconsistencies before training. The 14 exclusions described above were annotation-level removals resulting from this quality-control process and dental assessment; the image cohort itself was unchanged.

Two dentists reviewed all 2688 hold-out radiographs together with the corresponding ground-truth and final SBRF-Det prediction overlays. The review was performed after model evaluation and was used to characterize model behavior without altering reference annotations, split assignments, checkpoints, or numerical metrics. Correct multi-finding localizations and representative false-positive, false-negative, class-confusion, and box-localization errors were examined across the nine target categories. The successful and failure panels were selected purposively from this reviewed pool to cover predefined error types and to illustrate both reliable localization and clinically relevant residual errors. The panel selection was not random, prevalence-weighted, or intended to estimate the frequency of individual error modes. This qualitative assessment complements the quantitative analysis but was not designed as a formal reader-performance or interobserver-agreement study.

### 3.4. RP-P4 and SBRF-Det Architecture

#### 3.4.1. Network Architecture

The proposed detector introduces two successive levels of feature modeling at the P4/16 stage. The first, termed RP-P4, adapts representative feature interaction to the intermediate representation of a one-stage object detector. Its purpose is to mediate long-range communication through a compact representative space rather than through dense pairwise spatial interactions. The Gather–Interact–Distribute formulation is conceptually related to Representative Attention [30], while its detector-stage organization and use as the P4 feature-modeling pathway form the proposed RP-P4 configuration evaluated in this study.

**Figure 2:**
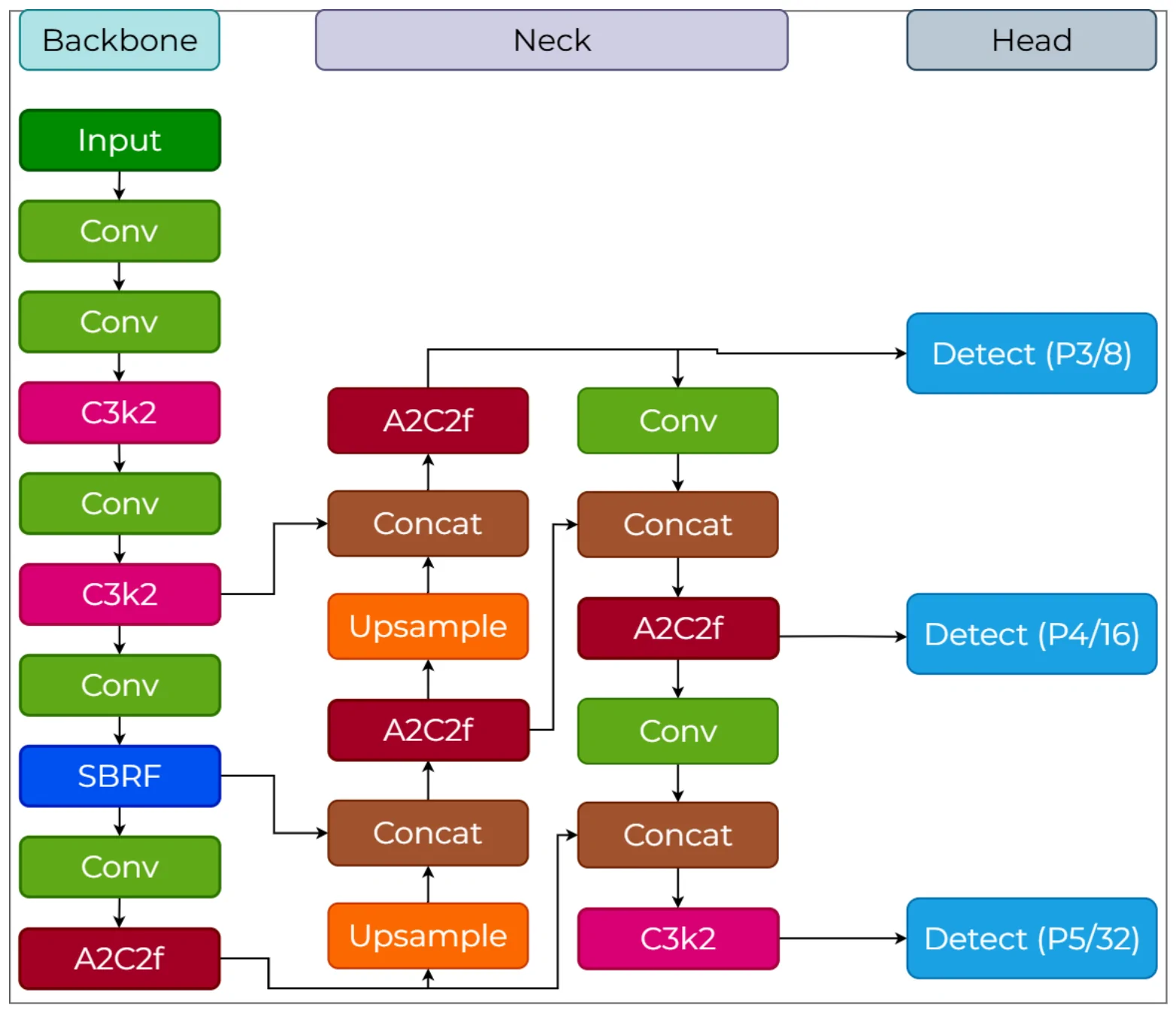
Architecture of SBRF-Det. The proposed feature-modeling pathway is placed at the P4/16 backbone stage, while the subsequent high-level backbone stage, the multi-scale neck, and the P3/8, P4/16, and P5/32 detection outputs are preserved.

SBRF-Det constitutes the complete extension of this pathway. It retains representative interaction but adds scale-dependent representative allocation, a local depth-wise structural pathway, and boundary-conditioned modulation. The surrounding backbone stages, multi-scale neck, and three-scale detection head are preserved so that the effect of the proposed P4 feature model can be examined without simultaneous redesign of the prediction architecture. Figure 2 summarizes the resulting network.

The P4/16 location was selected because it provides an intermediate representation in which local geometric information has not yet been excessively compressed, while semantic content is sufficiently developed for meaningful long-range interaction. With a 640 × 640 input, the P4 feature map is approximately 40 × 40 and contains 1600 spatial tokens. This scale is suitable for contextual reasoning across the panoramic field of view without discarding the fine structural evidence required for tight localization of small or weakly contrasted findings.

#### 3.4.2. SBRF Formulation

Let X denote the feature tensor entering SBRF, with batch size B, channel dimension C, and spatial dimensions H and W. A 1 × 1 input projection maps this tensor to the internal representation defined in Equation (3). This point-wise transformation changes the channel basis without altering spatial coordinates, so the representative, local, and boundary pathways remain aligned at each feature location.

(3) $$X_{p}=\varphi_{\mathrm{in}}\left(X\right),\;X_{p}\in R^{B\times C_{h}\times H\times W}$$

**Figure 3:**
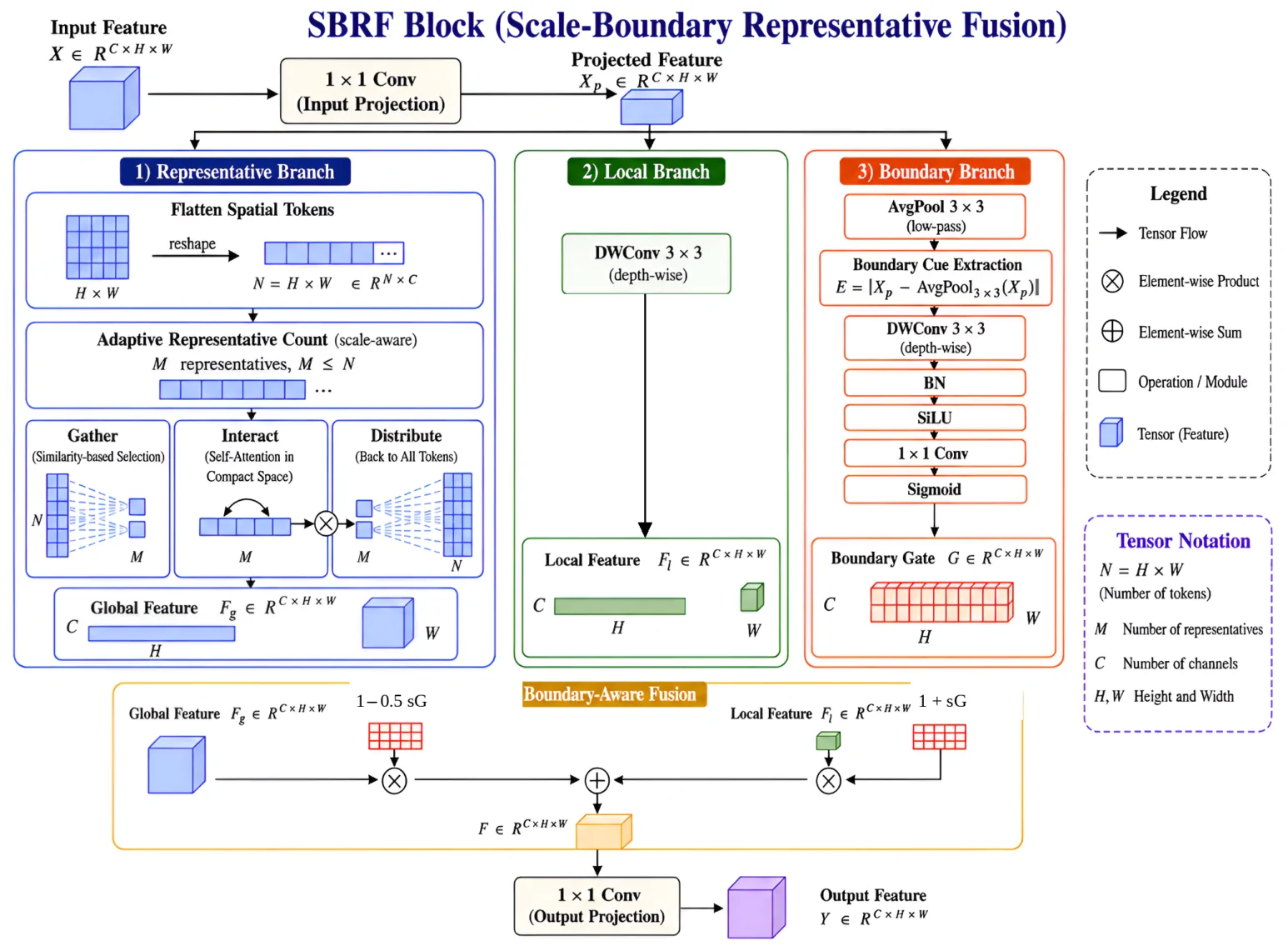
Internal organization of the Scale-Boundary Representative Fusion block. The representative branch performs Gather–Interact–Distribute communication, the local branch preserves short-range structure, and the boundary branch provides channel- and position-dependent modulation before output projection.

In Equation (3), the projected tensor uses the hidden channel dimension defined by the block configuration. The projected representation is processed by residual SBRF units within the surrounding cross-stage aggregation structure; intermediate responses are concatenated and compressed by a final point-wise projection. Before the detailed mathematical development, Figure 3 shows how the representative, local, and boundary pathways remain spatially aligned and are combined before output projection.

#### 3.4.3. Scale-Adaptive Representative Modeling

The representative branch first converts the projected feature map into the token sequence specified in Equation (4). The reshape preserves all H × W spatial positions while expressing them as N tokens that can be processed by the representative mechanism. No spatial averaging is performed at this step, which is important because the subsequent routing must still have access to fine P4-level structure.

(4) $$T=\mathrm{reshape}\left(X_{p}\right)\in R^{B\times N\times C_{h}},\;N=HW$$

The token sequence is not processed by direct all-to-all attention. Instead, the Gather–Interact–Distribute formulation follows Representative Attention [30], while SBRF makes the representative budget dependent on spatial scale according to Equation (5).

(5) $$M=\min\;\left(M_{\max},\max\;\left(M_{\min},\left\lceil \frac{N}{\tau }\right\rceil \right)\right),\;M\leq N$$

Under Equation (5), the minimum and maximum representative counts were set to 4 and 32, respectively, with 64 tokens assigned per representative. For the 40 × 40 P4 feature map used at the fixed 640 × 640 input resolution, N=1600 and Equation (5) gives M=25. Because the present P4 experiments use this single input resolution, they do not isolate the scale-dependent rule against a fixed-*M* control. Accordingly, Equation (5) is reported as an implemented architectural rule rather than as an independently proven source of the observed gain; fixed-*M* and cross-resolution sensitivity analyses are reserved for future work.

Point-wise projections generate the multi-head query, key, and value tensors in Equation (6). The projections place the same input representation into complementary subspaces for retrieval, semantic routing, and information transport. Splitting the channel dimension across attention heads further allows distinct representative relationships to be modeled in parallel without changing the spatial resolution of the feature map.

(6) $$Q=X_{p}W_{Q},\;K=X_{p}W_{K},\;V=X_{p}W_{V},\;d=\frac{C_{h}}{h}$$

In Equation (6), h is the number of attention heads and d is the per-head feature dimension. During Gather, learned per-head semantic anchors assign spatial keys to representative slots according to feature similarity, as formalized in Equation (7).

(7) $$A={\mathrm{Softmax}}_{M}\left(KW_{g}\right),\;A\in R^{B\times h\times N\times M}$$

The assignments are subsequently normalized over spatial tokens according to Equation (8). This second normalization controls the total contribution accumulated by each representative rather than allowing a representative to be dominated simply because many background tokens are routed to it. The operation is particularly relevant to panoramic radiographs, in which large homogeneous regions can otherwise outweigh small but diagnostically important structures.

(8) $${\hat{A}}_{n,m}=\frac{A_{n,m}}{\sum_{t=1}^{N}A_{t,m}+\varepsilon }$$

This normalization limits domination by large or repetitive background regions. Latent keys and values are then obtained through the weighted aggregation in Equation (9).

(9) $$K_{L}={\hat{A}}^{T}K,\;V_{L}={\hat{A}}^{T}V$$

Interaction among the *M* representatives is performed in the compact latent space following the latent interaction used in Representative Attention [30]. The gathered tensors are first normalized as ${\bar{K}}_{L}=\mathrm{LN}\left(K_{L}\right)$ and ${\bar{V}}_{L}=\mathrm{LN}\left(V_{L}\right)$. The latent self-attention queries, keys, and values are then projected from the normalized latent value tensor, so no additional undefined latent query tensor is assumed. The complete interaction is defined in Equation (10).

(10) $$\begin{array}{cc} {\tilde{Q}}_{L} & ={\bar{V}}_{L}W_{Q}^{L},\;{\tilde{K}}_{L}={\bar{V}}_{L}W_{K}^{L},\;{\tilde{V}}_{L}={\bar{V}}_{L}W_{V}^{L},\\ Z_{L} & ={\bar{V}}_{L}+\mathrm{Softmax}\;\left(\frac{{\tilde{Q}}_{L}{\tilde{K}}_{L}^{T}}{\sqrt{d}}\right){\tilde{V}}_{L}. \end{array}$$

Here, $K_{L},V_{L},{\bar{K}}_{L},{\bar{V}}_{L},{\tilde{Q}}_{L},{\tilde{K}}_{L},{\tilde{V}}_{L},$ and $Z_{L}$ have shape B×h×M×d, while $W_{Q}^{L}$, $W_{K}^{L}$, and $W_{V}^{L}\in R^{d\times d}$ are learned per-head latent projections shared across representative positions. Thus, ${\bar{K}}_{L}$ retains the routing geometry used in the subsequent distribution step, whereas ${\bar{V}}_{L}$ carries the contextual content refined by latent self-attention.

Finally, Distribute returns the refined semantic information to every spatial location through Equation (11). Spatial queries determine how strongly each location retrieves information from the latent representatives, restoring the global context to the original token grid. This step closes the Gather–Interact–Distribute cycle and produces a global response that remains spatially aligned with the local and boundary branches.

(11) $$O_{g}=\mathrm{Softmax}\;\left(\frac{Q{\bar{K}}_{L}^{T}}{\sqrt{d}}\right)Z_{L}$$

The resulting tensor is reshaped to the global representative feature. Because spatial communication is mediated by a bounded number of semantic representatives, distant regions that share similar radiographic characteristics can exchange information without constructing a full N-by-N attention matrix.

#### 3.4.4. Local Structure and Boundary Estimation

A representative bottleneck alone can weaken high-frequency information that is important for box placement. SBRF therefore retains a separate local path in which the value tensor is reshaped to the spatial domain and processed with the 3 × 3 depth-wise convolution defined in Equation (12). Channel-wise depth-wise filtering preserves short-range structural variation with limited additional computation and avoids replacing the global branch with another dense convolutional pathway.

(12) $$F_{l}={\mathrm{DWConv}}_{3\times 3}\left(\mathrm{reshape}\left(V\right)\right),\;F_{l}\in R^{B\times C_{h}\times H\times W}$$

This path preserves neighborhood-level structure with limited computational overhead. In parallel, the boundary cue is extracted from the projected feature map using the high-frequency residual in Equation (13). Subtracting the locally averaged response emphasizes feature transitions relative to their immediate neighborhood, which provides an explicit signal for margins, interfaces, and other spatial changes that can influence tight bounding-box placement.

(13) $$E=\left|X_{p}-{\mathrm{AvgPool}}_{3\times 3}\left(X_{p}\right)\right|$$

The absolute residual emphasizes rapid local changes in the learned feature space. It is processed by a depth-wise 3 × 3 convolution, batch normalization, SiLU activation, and a 1 × 1 channel projection followed by a sigmoid, yielding the boundary gate in Equation (14). The sigmoid constrains the modulation map to a stable [0,1] range while retaining channel-specific responses, allowing the gate to vary across both anatomical position and feature type.

(14) $$G=\sigma \;\left({\mathrm{Conv}}_{1\times 1}\;\left(\mathrm{SiLU}\;\left(\mathrm{BN}\;\left({\mathrm{DWConv}}_{3\times 3}\left(E\right)\right)\right)\right)\right),\;G\in {\left[0,1\right]}^{B\times C_{h}\times H\times W}$$

The resulting gate *G* is channel dependent and bounded between zero and one. It can therefore assign different local–global balances to different feature channels at the same anatomical position. The modulation strength is learned according to Equation (15).

(15) $$s=\sigma \left(\beta \right),\;\beta_{0}=-1$$

In Equation (15), β is initialized to −1, corresponding to an initial modulation strength of approximately 0.27. This conservative initialization keeps the early optimization trajectory close to the representative-attention response rather than imposing strong boundary weighting from the first iterations. The modulation can then increase or decrease through gradient-based learning when the training data support a different local–global balance.

#### 3.4.5. Boundary-Aware Fusion and Residual Refinement

The representative and local pathways are fused with the asymmetric boundary-dependent rule in Equation (16), rather than by a fixed element-wise sum. The formulation intentionally amplifies the local pathway more strongly than it suppresses the global pathway at high-gate locations, preserving semantic context while increasing sensitivity to structural transitions. This asymmetry is designed to avoid the failure mode in which boundary emphasis removes the contextual information needed to distinguish visually similar dental findings.

(16) $$F=F_{g}⊙\left(1-\frac{1}{2}sG\right)+F_{l}⊙\left(1+sG\right)$$

In Equation (16), the two feature terms denote the global representative response and the local depth-wise response, respectively, and the element-wise operator applies the learned gate at each channel and spatial position. A strong boundary response increases the local contribution while only moderately attenuating the global branch. The fused map is then projected according to Equation (17).

(17) $$Y=\varphi_{\mathrm{out}}\left(F\right)$$

Residual refinement is subsequently applied according to Equation (18). The first residual connection preserves the incoming representation while adding the SBRF response, and the second refines the result through the point-wise feed-forward transformation. This two-stage residual organization supports stable optimization and allows the proposed feature interaction to be introduced without discarding the representation learned by the surrounding backbone.

(18) $$X^{\prime }=X+A_{\mathrm{SBRF}}\left(X\right),\;X^{\prime \prime }=X^{\prime }+\mathrm{MLP}\left(X^{\prime }\right)$$

The feed-forward expansion ratio was 2.0 in the implemented SBRF block. The combination of representative context, local depth-wise filtering, and boundary-conditioned fusion addresses complementary error sources in panoramic radiographs. Global context helps distinguish visually similar structures that occur in different anatomical settings, while local evidence is retained around lesion margins, restoration interfaces, root structures, and other high-frequency regions. The P4 placement further preserves a spatial resolution at which these cues can influence box geometry. This design is particularly relevant to mAP50-95, where correct class prediction alone is insufficient and bounding-box overlap must remain accurate across progressively stricter IoU thresholds.

### 3.5. Detection Objective

For the controlled A0–A1–A2 architectural comparison, the three-scale prediction head jointly estimates class probabilities and bounding-box geometry using the common composite objective defined in Equation (19). The objective and loss weights were held constant across A0, RP-P4, and SBRF-Det so that the staged ablation did not introduce a simultaneous change in the optimization target. The broader 22-model experiment is interpreted as a common-framework benchmark rather than an optimizer- or objective-identical causal comparison across detector families, because the resolved family-specific training states were not uniformly recoverable from the available run records.

(19) $$L=\lambda_{\mathrm{box}}L_{\mathrm{box}}+\lambda_{\mathrm{cls}}L_{\mathrm{cls}}+\lambda_{\mathrm{dfl}}L_{\mathrm{DFL}}$$

The box, classification, and distribution-focal loss gains were 7.5, 0.5, and 1.5, respectively. Classification was optimized with binary cross-entropy with logits. Localization used Complete IoU (CIoU). For a predicted box and its matched ground-truth box, intersection over union is defined in Equation (20).

(20) $$\mathrm{IoU}\left(B_{p},B_{g}\right)=\frac{|B_{p}\cap B_{g}|}{|B_{p}\cup B_{g}|}$$

CIoU augments overlap with center-distance and aspect-ratio terms. The aspect-ratio discrepancy and its adaptive weighting are defined in Equations (21) and (22), respectively. These terms complement the overlap component by penalizing geometrically inconsistent boxes even when their intersection is relatively high, which is useful when precise localization is evaluated across strict IoU thresholds.

(21) $$v=\frac{4}{\pi^{2}}{\left(\arctan\frac{w_{g}}{h_{g}}-\arctan\frac{w_{p}}{h_{p}}\right)}^{2}$$

(22) $$\alpha =\frac{v}{1-\mathrm{IoU}+v}$$

These terms yield the CIoU loss given in Equation (23). The objective therefore combines overlap, center alignment, and aspect-ratio consistency in a single regression criterion. Minimizing this quantity encourages predicted boxes to match not only the presence of a target but also its spatial extent and geometry.

(23) $$L_{\mathrm{CIoU}}=1-\mathrm{IoU}+\frac{\rho^{2}}{c^{2}}+\alpha v$$

In Equation (23), ρ denotes the Euclidean distance between the centers of the predicted and ground-truth boxes, whereas *c* denotes the diagonal length of the smallest enclosing box. Distribution Focal Loss refines the discrete representation of box distances; for a continuous target between two neighboring bins, the loss is defined in Equation (24).

(24) $$L_{\mathrm{DFL}}=-\left(y_{r}-y\right)\log p_{y_{l}}-\left(y-y_{l}\right)\log p_{y_{r}}$$

Equation (24) encourages probability mass to concentrate around the continuous target instead of collapsing the prediction to a single coarse discrete bin. The interpolation between neighboring bins provides a smoother supervision signal for box-distance estimation and complements the geometric constraints imposed by CIoU.

### 3.6. Training Protocol and Implementation

Three protocol strata are reported separately. For the 22-model screening benchmark, standard detectors and the corresponding A0–A2 configurations were trained under a common args.yaml-based policy, with architecture definition as the model-specific element; images were resized to 640 × 640 pixels, batch size was 16, and the training budget was 300 epochs. No additional dataset-specific intensity normalization was configured beyond the default preprocessing of Ultralytics 8.4.121. Automatic mixed precision and eight data-loading workers were used, deterministic execution was enabled, and mosaic augmentation was disabled for the final ten epochs. The B1–B3 component-removal runs and their matched A2 reference used the predefined 300-epoch, 640 × 640, batch-4, seed-0 protocol recorded in their checkpoints. The multi-seed robustness analysis used three independently trained runs for each A0/A1/A2 configuration, with a distinct random seed assigned to each run. The seed identities were verified in the training records, allowing the resulting mean and standard deviation to be interpreted as between-seed variability rather than as an unlabeled repeated-run summary.

The stored training configurations for the nine A0/A1/A2 robustness checkpoints were examined directly. All nine used a 300-epoch budget, batch size 16, imgsz=640, rect=False, deterministic execution, patience 20, and three warm-up epochs. The numerical seeds were A0: 1, 2, and 4; A1: 2, 3, and 4; and A2: 0, 1, and 2. The checkpoint metadata field reported epoch 300 for each selected file. These records separate the intended random-seed variation from other training factors and provide the exact batch and early-stopping settings used in the robustness analysis.

The stored training arguments specified an initial learning-rate parameter of 0.01, a final learning-rate factor of 0.01, momentum of 0.937, weight decay of $5\times 10^{-4}$, three warm-up epochs, a warm-up momentum of 0.8, and a warm-up bias learning rate of 0.1. Optimizer selection was set to auto. Inspection of the Ultralytics 8.4.121 optimizer builder showed that runs exceeding 10,000 optimization iterations resolve this policy to MuSGD with a learning rate of 0.01 and a momentum of 0.9; stored run metadata independently identified MuSGD for the examined long-run training records. The value 0.937 is therefore the stored user-level momentum argument, whereas 0.9 is the resolved MuSGD momentum under the automatic policy. The final learning-rate factor and weight-decay policy remained those recorded in the run arguments. The geometric and photometric augmentation policy was also fixed: HSV hue, saturation, and value perturbations were 0.015, 0.7, and 0.4; translation was 0.1; scale augmentation was 0.5; and horizontal flipping was applied with a probability of 0.5. Rotation, vertical flipping, shear, perspective transformation, MixUp, CutMix, and copy–paste augmentation were disabled. Horizontal reflection was retained because it preserves the superior–inferior organization of a panoramic radiograph while exchanging anatomically plausible left–right positions. Vertical flipping and stronger geometric warps were disabled because they can create anatomically implausible orientations or spatial relationships. The photometric perturbations were used as appearance regularization for acquisition/display variability and were not interpreted as a physical simulation of radiographic formation. Pretrained weights were obtained from the official Ultralytics distribution for the corresponding architecture where available; this constitutes family-matched pretraining rather than identical tensor initialization across different detector families. The A0–A2 pathway is based on the YOLO12l checkpoint lineage, and the B1–B3 component-removal runs were initialized from yolo12l.pt, as recorded in the predefined training protocol. For the multi-seed runs, the framework-generated best.pt checkpoint was used for evaluation, with checkpoint selection governed by the validation procedure of Ultralytics 8.4.121; the hold-out cohort was not used to choose checkpoints. The random seed was the intended source of stochastic variation in this analysis.

A tensor-transfer analysis quantified how much of the YOLO12l initialization could be inherited after the architectural substitutions. For A0, all 26,450,784 parameter elements included in the transfer analysis were shape compatible with the pretrained checkpoint (100%). A1 inherited 26,346,336 of 26,423,136 elements (99.7093%), leaving 76,800 architecture-specific elements without a pretrained shape match. A2 inherited 26,346,336 of 27,004,776 elements (97.5618%), leaving 658,440 architecture-specific elements. The unmatched elements are therefore described as architecture-specific/native initialization rather than being assigned an unverified random-initialization label.

The primary architectural ablation used three matched configurations. A0 was the unmodified large reference detector, A1 introduced the RP-P4 pathway at P4/16, and A2 used the complete SBRF formulation at the same location. All remaining backbone, neck, loss, and detection-head settings were matched within the controlled comparison. A controlled multi-seed robustness analysis evaluated three independently trained runs with distinct random seeds for each A0/A1/A2 configuration. Mean and SD across these runs quantify between-seed variability in the principal detection metrics. The broader 22-model benchmark is reported separately from this multi-seed analysis.

Feature-scale sensitivity was assessed by placing the complete SBRF feature-modeling block at P3/8, P4/16, or P5/32 while holding the representative count fixed at M=25 for all three placements. The P4 condition used the reference A2 checkpoint, for which the scale-dependent rule also yields M=25 at 640×640 input, whereas the P3 and P5 variants were trained with the same 300-epoch, seed-0, batch-4, pretrained-YOLO12l protocol used for the controlled component study. Fixing *M* prevents automatic representative-count changes from being conflated with feature-scale location, although moving the block necessarily changes the surrounding stage context. Fusion sensitivity was evaluated at P4 with M=25 by replacing the asymmetric rule in Equation (16) with the gated additive form $F_{\mathrm{add}}=F_{g}+\left(sG\right)⊙F_{l}$ while retaining the same training and evaluation protocol. These feature-scale and fusion comparisons are interpreted as controlled single-run sensitivity analyses rather than multi-seed population estimates.

All primary hold-out evaluations used imgsz=640 with rect=False. The framework preserved the native aspect ratio and applied letterbox padding to a square 640×640 tensor; images were not stretched to fill the square. Native width and height were read directly from every hold-out image during the geometry analysis, and no single native image size was assumed. A runtime feature hook on the reference A2 checkpoint confirmed a 40×40 P4 tensor, so N=1600 and Equation (5) yields M=25 for the evaluated P4 configuration. Quantitative claims in this study are consequently restricted to the 640×640 setting.

Experiments were executed on a workstation equipped with an Intel Core Ultra 7 processor, 64 GB system memory, and an NVIDIA GeForce RTX 5090 GPU with approximately 32 GB of video memory. The software environment comprised Ubuntu 24.04, Python 3.14.3, PyTorch 2.10.0 with CUDA 12.8 support, and Ultralytics 8.4.121. The same environment was used for training and final evaluation of all models.

### 3.7. Evaluation Metrics and Efficiency Analysis

Final performance was measured exclusively on the hold-out test set of 2688 radiographs and 18,639 annotated instances. Precision and recall were computed according to Equation (25). These complementary measures quantify false-positive control and target recovery, respectively, and were reported together because the clinical relevance of a detector cannot be characterized by either error type alone.

(25) $$P=\frac{TP}{TP+FP},\;R=\frac{TP}{TP+FN}$$

In Equation (25), TP, FP, and FN denote true-positive, false-positive, and false-negative detections. For class *c* at IoU threshold *t*, Average Precision was obtained from the area under the precision–recall curve as defined in Equation (26).

(26) $$AP_{c,t}=\int_{0}^{1}P_{c}\left(R_{c},t\right)\;dR$$

The mean AP at IoU = 0.50 was calculated using Equation (27). This metric averages class-level AP under a permissive overlap threshold and is useful for comparing broad detection capability across the nine findings. It was retained for continuity with the dental object-detection literature, but it was not treated as the sole criterion for ranking models.

(27) $$mAP_{50}=\frac{1}{K}\sum_{c=1}^{K}AP_{c}\left(0.50\right),\;K=9$$

In Equation (27), K=9 classes. The primary localization metric was the COCO-style average over ten IoU thresholds from 0.50 to 0.95 in steps of 0.05, as defined in Equation (28).

(28) $$mAP_{50:95}=\frac{1}{10K}\sum_{t\in \{0.50,0.55,\ldots ,0.95\}}\sum_{c=1}^{K}AP_{c,t}$$

mAP50-95 was treated as the principal accuracy criterion because it penalizes loose localization that may still be counted as correct at IoU = 0.50. Class-specific AP values were also examined to determine whether aggregate performance was dominated by frequent or structurally easier categories. During validation, the maximum number of detections per image was 300 and the IoU setting used by the postprocessing configuration was 0.70.

Computational efficiency was characterized by trainable parameter count, floating-point operations at 640 × 640 input resolution, and batch-1 runtime on the RTX 5090 GPU. For the focal A0, A1, A2, B1, B2, and B3 variants, a dedicated final profiling pass was performed under one fixed computational environment. Each model first completed one unrecorded warm-up validation pass, followed by three measured batch-1 passes over the same hold-out cohort. The framework-reported preprocessing, inference, and postprocessing times were recorded separately and summed to obtain validation-pipeline time per image. Pure-inference throughput was calculated as $1000/t_{\inf}$, and pipeline throughput as $1000/(t_{\mathrm{pre}}+t_{\inf}+t_{\mathrm{post}})$. Peak CUDA memory allocation was recorded during the same profiling runs. These quantities characterize computational execution of the detector and should not be interpreted as complete chair-side workflow time, which would also depend on image acquisition, transfer, visualization, and user interaction.

### 3.8. Statistical Analysis

For multi-seed robustness analysis, A0, A1, and A2 were each summarized across three independently trained runs initialized with distinct random seeds using the arithmetic mean and sample standard deviation of mAP50 and mAP50-95. The resulting SD values quantify between-seed variability under the reported training protocol. A paired nonparametric hold-out bootstrap with 10,000 resampling iterations was additionally applied to the three-seed mean performance. Because explicit patient identifiers are not available in the distributed metadata, patient-clustered resampling could not be reconstructed and is not claimed. Instead, exact-content duplicate groups were defined using the same hash-based grouping procedure; all 2688 hold-out images were unique, so each radiograph formed a singleton resampling unit. For each bootstrap iteration, identical resampled hold-out units were applied to A0, A1, and A2, and the comparison statistic was the difference in mean mAP50-95 across the three seed-specific runs of each model. Percentile-based 95% intervals were calculated for A1–A0, A2–A0, and A2–A1. The multi-seed summaries quantify training variability, whereas the paired bootstrap intervals quantify radiograph-level uncertainty in the corresponding three-seed mean differences; neither analysis is interpreted as patient-level clinical uncertainty. Calibration values from the single-run benchmark are reported descriptively and are not used to claim clinical threshold optimality.

Two secondary localization analyses complemented the primary three-seed uncertainty analysis. First, the reference A0 and A2 checkpoints were compared with 1000 paired radiograph-level bootstrap resamples for class-specific mAP50-95 differences and COCO-style small, medium, and large object strata. These intervals are reported separately from the 10,000-resample three-seed analysis because they address a different, checkpoint-level question. Second, true-positive localization geometry was summarized using greedy same-class matching at confidence ≥ 0.25 and IoU ≥ 0.50. Because A0 and A2 can produce different numbers of matched detections, these IoU distributions are descriptive model-specific summaries rather than a one-to-one paired test of identical ground-truth objects.

### 3.9. Computational Characteristics of SBRF

For the instantiated P4 SBRF block, the incoming stage tensor has 512 channels and the internal projected width is $C_{h}=256$. The analytical expressions below therefore use the actual hidden-channel width of the implemented block. They describe asymptotic arithmetic structure and are not equated with the framework-reported GFLOP total, which does not capture all tensor-interaction and element-wise execution costs in the same way as an operator-level runtime profile.

Dense spatial attention forms pairwise interactions among N tokens and therefore introduces a term that scales quadratically with the number of spatial tokens. SBRF instead communicates through M latent representatives, with M bounded by 32. Ignoring lower-order normalization and activation terms, the dominant representative-path cost is expressed in Equation (29).

(29) $$\Omega_{\mathrm{rep}}=4NC_{h}^{2}+5NMC_{h}+2M^{2}C_{h}+k^{2}NC_{h}$$

In Equation (29), the first term represents point-wise feature projections, the mixed token-representative terms arise from Gather and Distribute, the latent interaction is quadratic only in the bounded representative count, and k = 3 denotes the local depth-wise kernel size. The boundary pathway adds the approximate cost in Equation (30).

(30) $$\Omega_{\mathrm{boundary}}=k^{2}NC_{h}+NC_{h}^{2}+O\left(NC_{h}\right)$$

Combining the representative and boundary pathways gives the core SBRF complexity approximation in Equation (31). The expression makes explicit that the latent interaction depends on the bounded representative count M rather than a dense N × N token-affinity matrix. Consequently, the additional boundary-aware processing changes the constant and linear terms of the block while preserving the intended scalable global-interaction structure.

(31) $$\Omega_{\mathrm{SBRF}}\approx 5NC_{h}^{2}+5NMC_{h}+2M^{2}C_{h}+2k^{2}NC_{h}+O\left(NC_{h}\right)$$

Because M remains small relative to N, the representative interaction avoids restoring a dense N-by-N affinity matrix. The additional boundary route is composed primarily of depth-wise filtering, a point-wise projection, and element-wise modulation. The module therefore increases feature selectivity at the intermediate backbone stage without relying on a disproportionate increase in network depth or width.

## 4. Results

### 4.1. Overall Benchmark Performance

The complete released hold-out benchmark is reported in Table 4. It contains 20 standard configurations from the YOLOv10, YOLO11, YOLO12, and YOLO26 families together with the RP-P4 pathway and SBRF-Det. Every entry was evaluated on the same 2688-image hold-out cohort using the common protocol described in Section 3.

SBRF-Det ranked first for recall (0.8979), mAP50 (0.9152), and mAP50-95 (0.7029). Precision was highest for YOLO12m (0.8818), indicating that the proposed configuration did not dominate every metric. The second-highest strict-localization result was obtained by RP-P4 (0.7009), followed by YOLO12l (0.6934), YOLO11l (0.6933), and YOLO11m (0.6924). Relative to the matched YOLO12l baseline, SBRF-Det increased mAP50-95 by 0.95 percentage points, mAP50 by 0.76 points, and recall by 1.08 points, while precision was 0.08 points lower. The proposed model used 26.90 M parameters, approximately 2.11% more than YOLO12l, while GFLOPs decreased by 3.06% from 95.13 to 92.22. Batch-1 inference latency increased from 6.07 to 8.56 ms/image, showing that arithmetic complexity and wall-clock execution are not interchangeable measures for representative routing.

### 4.2. Accuracy-Efficiency Trade-Offs

**Figure 4:**
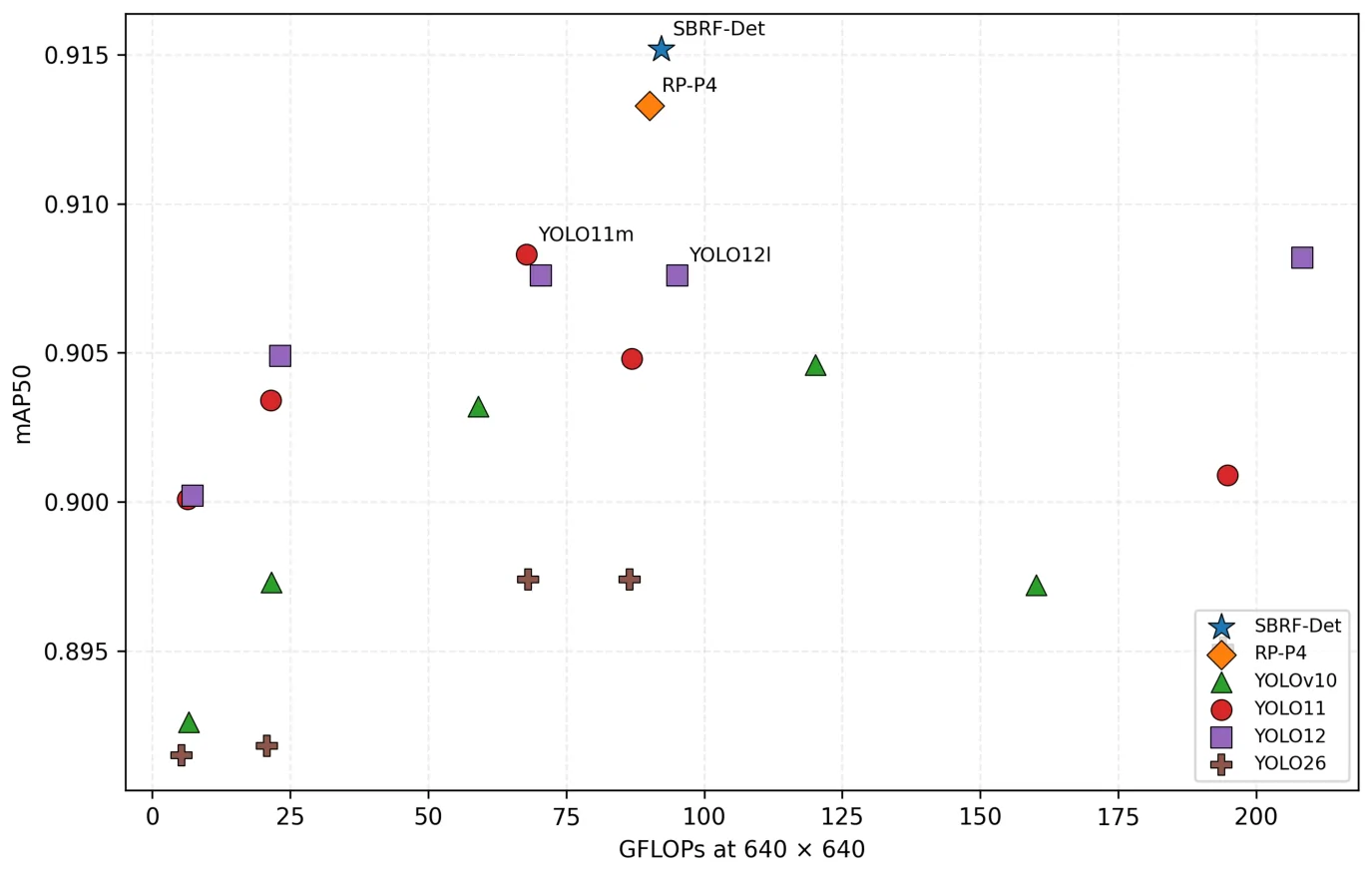
GFLOPs versus mAP50 for the 22 evaluated configurations. SBRF-Det denotes the complete A2 configuration, while RP-P4 denotes the A1 representative pathway.

The relationship between arithmetic complexity and detection accuracy is visualized in Figure 4. Each marker represents one evaluated configuration; color and shape identify the model family, while SBRF-Det and RP-P4 are highlighted separately.

The highest mAP50 was not obtained by the most computationally expensive network. SBRF-Det reached 0.9152 mAP50 at 92.22 GFLOPs, whereas extra-large configurations exceeding 190 GFLOPs remained below 0.909. RP-P4 occupied a similar high-accuracy region with lower arithmetic cost (90.15 GFLOPs) and 0.9133 mAP50. YOLO11m provided a strong lower-cost operating point at 67.80 GFLOPs and 0.9083 mAP50. Within this benchmark, the proposed configurations occupied a favorable accuracy–complexity region without requiring the largest model scale.

**Figure 5:**
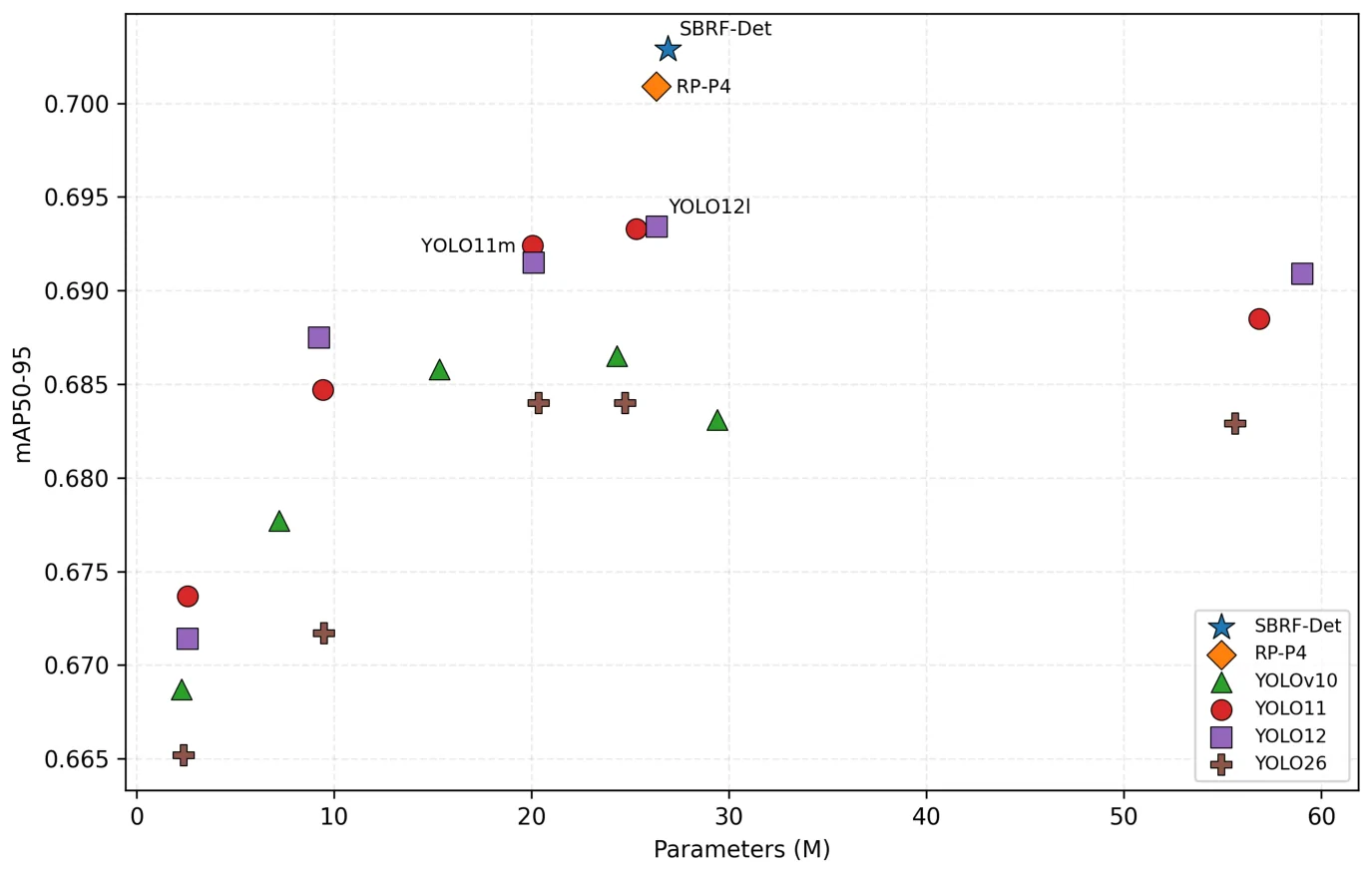
Parameter count versus mAP50-95 for all evaluated configurations. SBRF-Det denotes the complete A2 configuration.

Parameter efficiency under the stricter localization metric is shown in Figure 5. This view is particularly informative because mAP50-95 penalizes imprecise boxes across progressively higher IoU thresholds.

SBRF-Det and RP-P4 form the upper envelope of the parameter-accuracy distribution. Both use approximately 26 M parameters yet exceed extra-large models with 55–59 M parameters. Among standard models, YOLO12l and YOLO11l reach 0.6934 and 0.6933 mAP50-95, respectively, while YOLO11m offers a competitive 0.6924 with only 20.04 M parameters and approximately 2.95 ms/image inference. SBRF-Det requires 8.56 ms/image, so its principal advantage is localization accuracy at moderate model size rather than minimum latency.

### 4.3. Progressive and Component-Level Ablation of the Proposed P4 Feature Model

Table 5 presents the controlled A0–A1–A2 ablation used to quantify the two proposed stages of feature modeling. The P4/16 location, training protocol, data partitions, and evaluation settings were fixed across the three configurations.

Introducing the RP-P4 pathway increased mAP50-95 from 0.6934 to 0.7009, corresponding to a gain of 0.75 percentage points over the matched baseline. Extending RP-P4 with the complete SBRF formulation increased the score further to 0.7029, yielding a total gain of 0.95 percentage points over A0. Recall increased from 0.8871 to 0.8965 and then 0.8979, while mAP50 increased from 0.9076 to 0.9133 and 0.9152. The decomposition therefore shows that the detector-specific representative pathway contributes the largest initial gain, while scale-boundary fusion provides a further refinement. The comparatively smaller A1-to-A2 increment is reported conservatively and is not interpreted as evidence of pairwise statistical superiority in isolation.

Uncertainty is reported in dedicated analyses rather than duplicated across the descriptive benchmark tables: Section 4.4 provides checkpoint-level class-specific and size-stratified A0–A2 intervals, whereas Section 4.5 reports the three-seed summaries and paired 10,000-resample A0–A1–A2 intervals. This separation avoids mixing single-checkpoint and multi-seed uncertainty estimates while keeping the principal results in the main text.

A controlled internal-pathway analysis removed the three components of the complete A2 formulation one at a time while retaining the 300-epoch training budget, seed 0, 640 × 640 input, batch size 4, pretrained YOLO12l initialization, optimizer policy, learning-rate settings, augmentation policy, and hold-out evaluation protocol. A complementary sensitivity analysis then examined feature-scale placement at a fixed representative count of M=25 and compared the native asymmetric fusion rule with a gated additive alternative at P4. Table 6 reports both analyses.

Removing any of the three internal pathways reduced strict localization relative to the complete A2 formulation. The largest observed reduction followed the removal of boundary-conditioned processing (−0.689 percentage points in mAP50-95), followed by the removal of representative modeling (−0.440 points) and the local structural pathway (−0.317 points). mAP50 and recall also remained highest for the complete formulation. The B2 result is informative because its parameter count remained close to A2 while strict localization still decreased, indicating that the observed A2 advantage cannot be explained solely by a larger parameter budget. These are controlled single-run removal effects and are not treated as population-level estimates of the relative importance of individual components.

With *M* fixed at 25, the native P4/16 configuration produced the highest strict-localization score among the three tested feature scales, reaching 0.7027 mAP50-95 and 0.7297 AP75, compared with 0.6912/0.7255 at P3/8 and 0.6900/0.7237 at P5/32. At the same P4 location and representative count, the gated additive fusion alternative reached 0.6916 mAP50-95 and 0.7240 AP75, whereas the native asymmetric fusion reached 0.7027 and 0.7297. These single-run sensitivity results provide empirical support for the selected P4 location and native fusion rule under the evaluated protocol, but they do not establish a population-level ranking or isolate the effect of adaptive representative count.

To place these accuracy changes alongside computational cost, all focal variants were re-profiled together using the dedicated batch-1 protocol described in Section 3.7. Table 7 reports the final controlled efficiency profile. The two complexity tables use different profiling states by design: the original 22-model screening script called model.fuse() before model.info(), whereas the final controlled lock counted parameters from the loaded checkpoint graph and recomputed FLOPs without an explicit pre-profile fusion step. The small differences in Params/GFLOPs between Table 4, Table 5 and Table 7 therefore reflect profiling convention/state rather than different training runs. Direct computational comparisons among A0–A2 and B1–B3 use the single convention in Table 7.

The final profile confirms that SBRF-Det does not obtain its localization gain by minimizing runtime. Relative to A0, A2 reduced the re-profiled arithmetic cost from 95.99 to 93.06 GFLOPs but increased pure inference time from 5.946 to 8.851 ms/image and validation-pipeline time from 6.618 to 9.540 ms/image. This corresponds to the pipeline throughput of 151.1 images/s for A0 and 104.8 images/s for A2. Over 2688 hold-out radiographs, the same per-image measurements correspond to approximately 17.8 s and 25.6 s of validation-pipeline processing, respectively. Peak allocated GPU memory increased from 228 MB for A0 to 333 MB for A2. The component variants further illustrate that arithmetic complexity, memory use, and wall-clock execution do not move in lockstep: B1 is the fastest removal variant, whereas B2 preserves almost the full A2 parameter and GFLOP budget while remaining slightly faster. The efficiency evidence therefore supports a localization–runtime trade-off rather than universal computational superiority.

An operator-level CUDA profile further explains the divergence between framework GFLOPs and measured latency. Across 30 profiled forward passes, total aten::conv2d time was similar for A0 and A2 (85.013 and 85.801 ms, respectively), whereas aten::bmm increased from 19.006 to 35.525 ms. A2 additionally contained 24.630 ms of aten::einsum activity and 11.844 ms of aten::matmul; A0 recorded 20.404 ms of aten::matmul. The bmm increase was approximately 86.9%. Because profiler entries can be nested, these event totals are not summed to estimate end-to-end latency. Instead, they show that representative interaction introduces tensor operations that are incompletely reflected by the convolution-oriented GFLOP summary, consistent with the independently measured wall-clock latency increase.

### 4.4. Class-Specific Detection and Localization Performance

Class-specific performance of SBRF-Det is summarized in Table 8. The same predefined 2688-image hold-out cohort was used for every class-level measurement.

At IoU 0.50, AP exceeded 0.98 for wisdom tooth, implant, porcelain crown, and ceramic bridge. Their mAP50-95 values were 0.8172, 0.8401, 0.8842, and 0.8766, respectively. Apical periodontitis remained the most difficult category (0.4526 mAP50-95), followed by decay (0.5303) and missing tooth (0.6050). The class-level results therefore show substantial heterogeneity across both pathological and treatment-related findings, and the aggregate detector score should not be interpreted as uniform performance across the nine categories.

A pathology-only summary was additionally calculated for apical periodontitis and decay, the two labels that represent active pathological processes in the nine-class task. Their mean AP50 increased from 0.7458 for A0 to 0.7604 for A2 (+1.46 percentage points), AP75 from 0.5082 to 0.5142 (+0.60 points), and mAP50-95 from 0.4760 to 0.4916 (+1.57 points). Thus, the direction of change was positive within the pathology subset, but its absolute strict-localization level remained substantially below the nine-class headline metric. The paired class-specific intervals in Table 8 further show that the strongest checkpoint-level evidence was concentrated in apical periodontitis, decay, wisdom tooth, dental filling, and root-canal filling, whereas intervals for missing tooth, implant, porcelain crown, and ceramic bridge included zero in this 1000-resample secondary analysis. For the size strata, only the medium-object interval excluded zero.

### 4.5. Multi-Seed Robustness and Paired Hold-Out Resampling

Across three independently trained runs with distinct random seeds for each configuration, A0, A1, and A2 achieved mean mAP50-95 values of 0.6909 ± 0.0012, 0.6957 ± 0.0005, and 0.6992 ± 0.0031, respectively (Table 9). The corresponding mAP50 means were 0.9055 ± 0.0025, 0.9100 ± 0.0013, and 0.9128 ± 0.0023. The ordering observed in the matched single-run ablation was therefore preserved across the three-seed analysis, providing direct evidence of robustness to random-seed variation.

**Figure 6:**
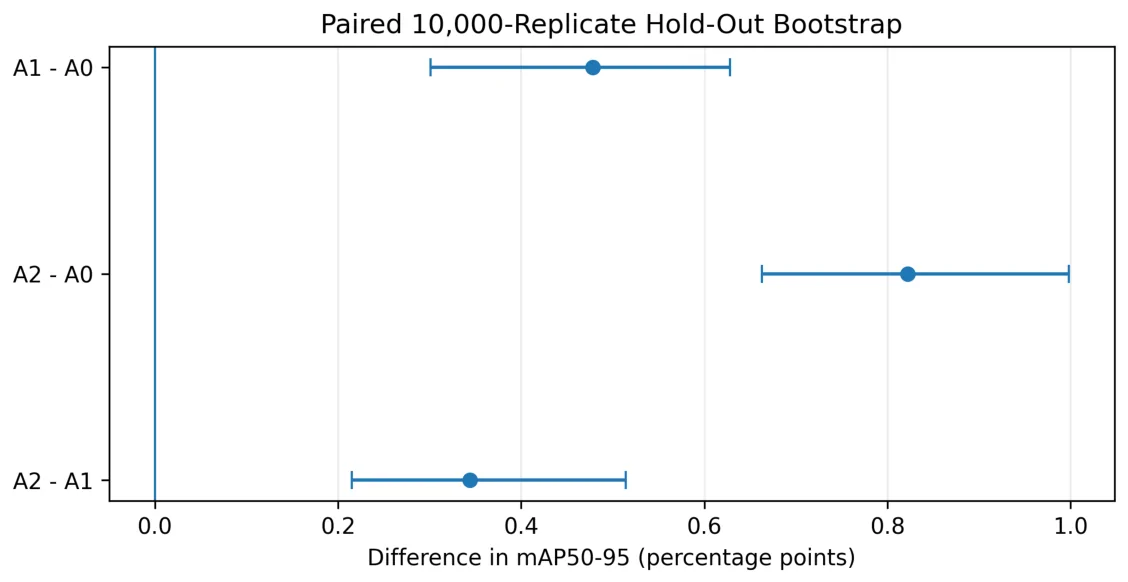
Paired 10,000-iteration radiograph-level bootstrap differences in three-seed mean mAP50-95. Points denote observed differences in percentage points and horizontal bars denote percentile-based 95% intervals.

All three paired intervals excluded zero under radiograph-level resampling of the three-seed mean predictions (Figure 6). The A2–A1 increment remained smaller than the total A2–A0 difference and is therefore interpreted as a modest refinement. These intervals complement the between-seed mean and SD estimates but do not establish patient-level uncertainty because the public release does not expose the identifiers required for patient-clustered resampling.

Calibration did not follow the same ranking as strict localization in the original single-run analysis: YOLO11l had the lowest ECE (0.0271) and Brier score (0.0376), whereas SBRF-Det yielded ECE 0.0385 and Brier 0.0420. The proposed model is therefore not presented as universally superior across calibration and runtime criteria.

### 4.6. Precision–Recall Behavior

**Figure 7:**
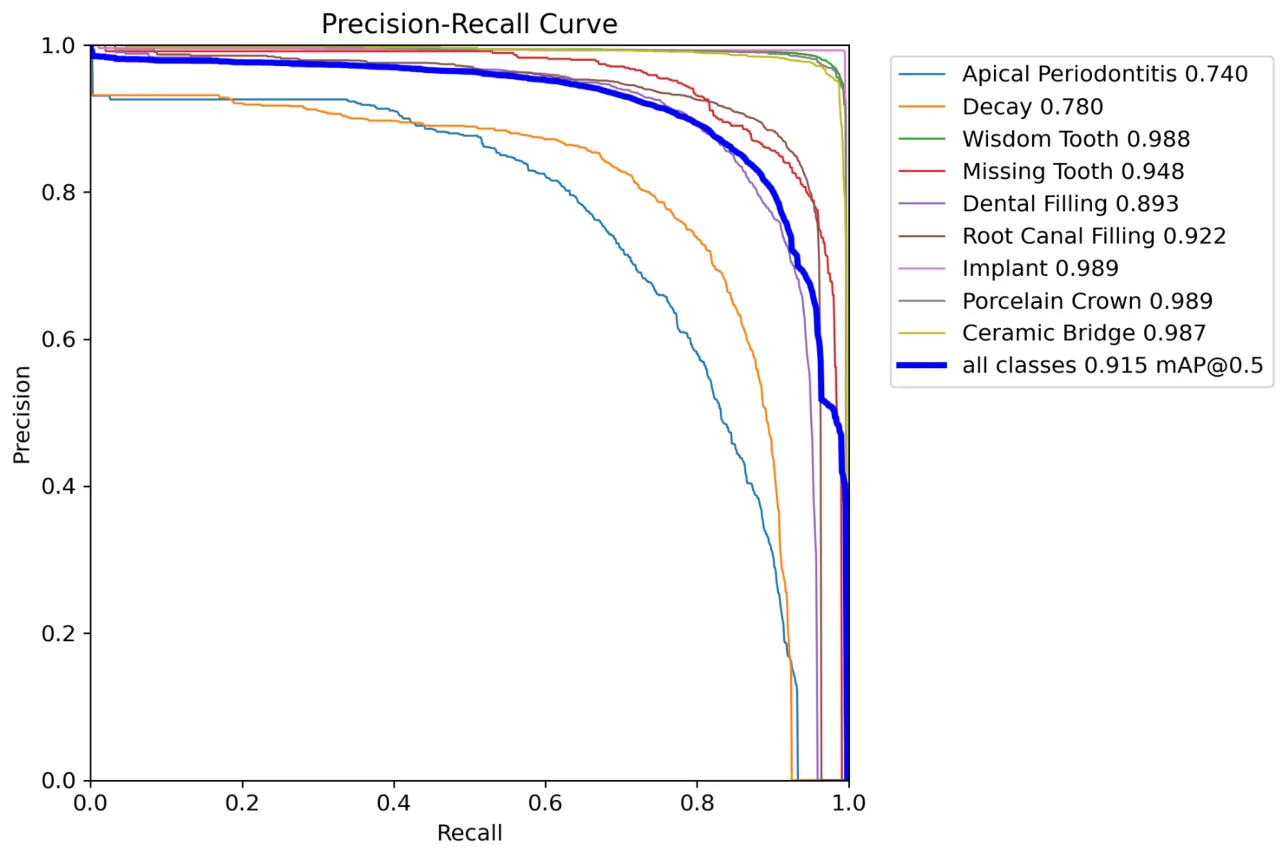
Precision–recall curves of SBRF-Det on the hold-out test set. The thick blue curve denotes the mean AP at IoU 0.50 across the nine categories.

The class-wise precision–recall curves of SBRF-Det are shown in Figure 7. The curve set provides a threshold-independent view of class separability and complements the class-level metrics in Table 8.

The mean AP at IoU 0.50 was 0.915. Implant and porcelain crown each reached approximately 0.989 AP50, wisdom tooth reached 0.988, and ceramic bridge reached 0.987. Apical periodontitis (0.740) and decay (0.780) produced the lowest curves, consistent with their lower strict AP values and the difficulty of subtle or low-contrast findings. The broader separation among classes confirms that the aggregate score does not conceal substantial category-specific heterogeneity.

### 4.7. Dentist-Reviewed Qualitative Performance and Error Analysis

**Figure 8:**
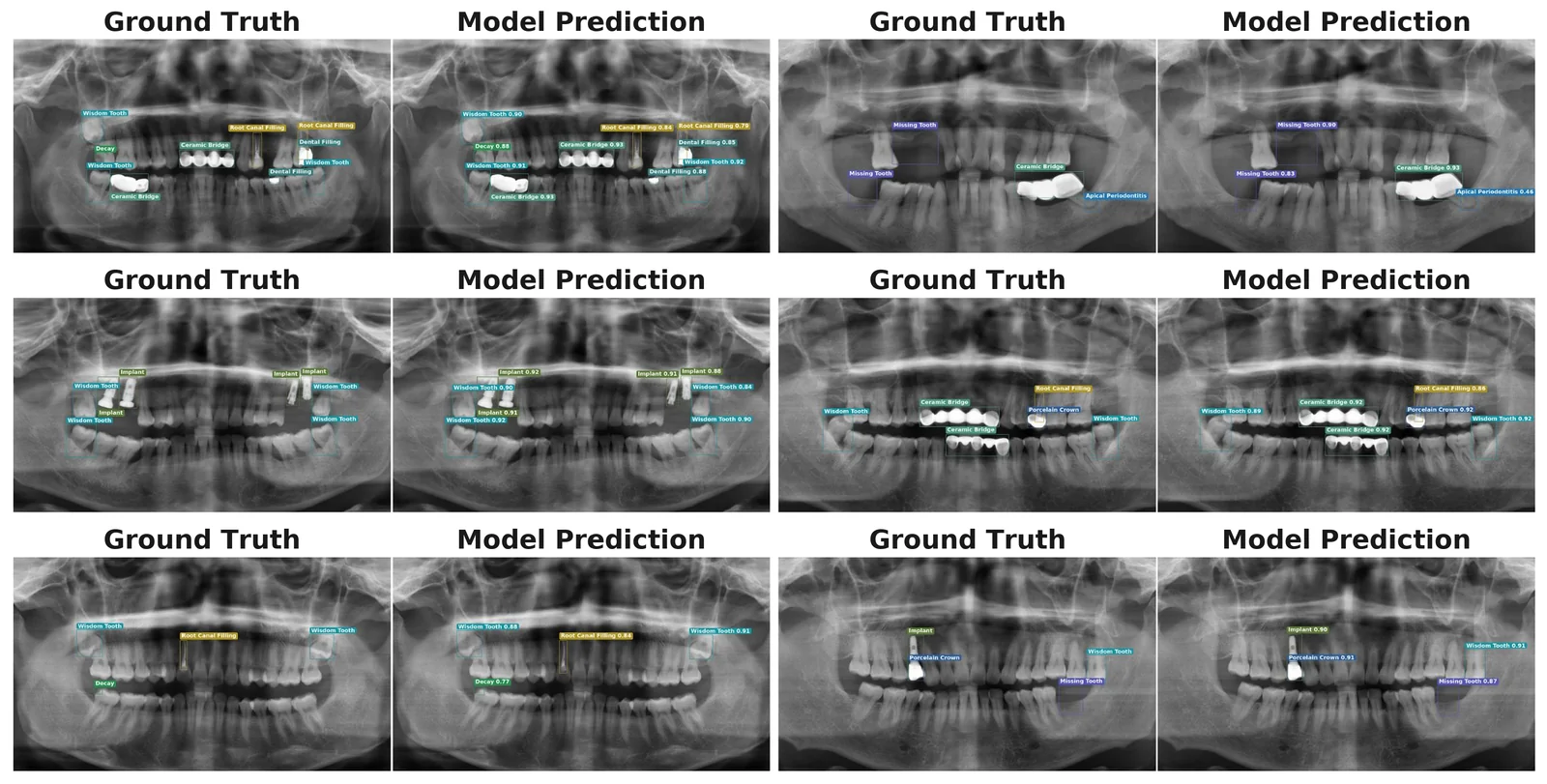
Dentist-reviewed successful multi-finding detections from the hold-out cohort. Ground-truth annotations and SBRF-Det predictions are shown side by side. The six cases collectively represent all nine target categories and illustrate simultaneous localization of pathological, anatomical-status, and treatment-related findings.

Six representative successful cases reviewed by two dentists are shown in Figure 8. Each panel pairs the reference annotations with the corresponding SBRF-Det output, and the cases collectively include all nine target categories.

Figure 8 shows close spatial correspondence for many structurally well-defined findings, including implants, porcelain crowns, ceramic bridges, root-canal fillings, dental fillings, and multiple wisdom teeth. Several radiographs contain three or more concurrent classes, illustrating successful simultaneous localization of spatially separated findings within the same examination. In addition, class-specific predictions can occupy the same or immediately adjacent tooth region without a mutually exclusive label constraint, which is consistent with the box-level YOLO formulation described in Section 3.1. Because tooth-instance identifiers are not part of the released annotations, these examples are interpreted qualitatively rather than as a tooth-ID-based coexistence rate. Successful examples also include decay, missing tooth, and apical periodontitis, although confidence and box agreement are more variable for these less conspicuous findings than for radiopaque prosthetic structures. This pattern is consistent with Table 8: porcelain crown (0.8842 mAP50-95), ceramic bridge (0.8766), and implant (0.8401) were among the strongest classes, whereas decay (0.5303) and apical periodontitis (0.4526) remained substantially more difficult.

**Figure 9:**
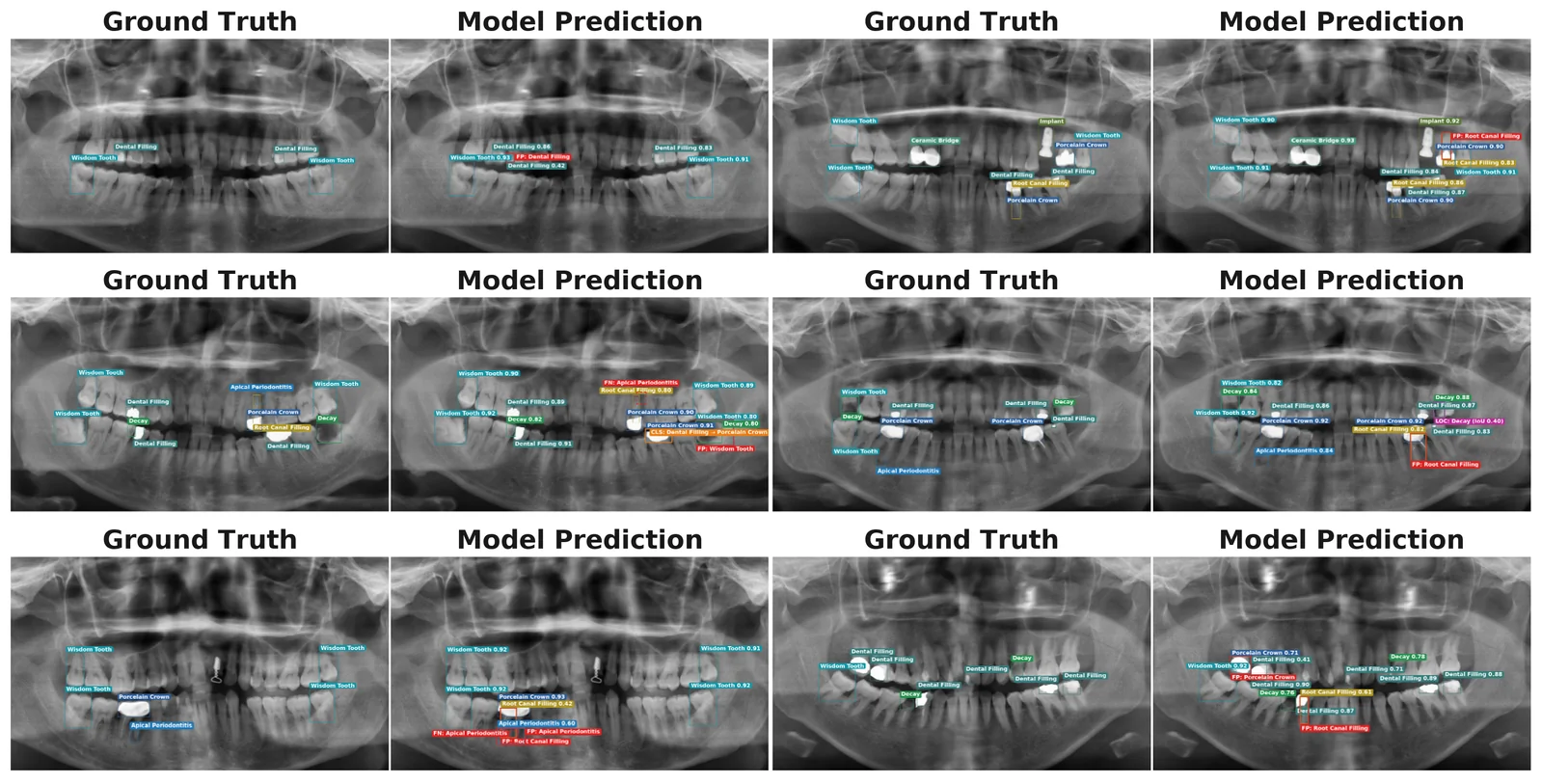
Dentist-reviewed representative failure cases from the hold-out cohort. Ground truth and model prediction are shown side by side. Red callouts identify false-positive (FP) and false-negative (FN) detections; orange callouts identify class-confusion (CLS) events. Low-confidence detections and imperfect box alignment are also visible where present.

Figure 9 complements the successful examples with six dentist-reviewed cases selected to represent distinct error modes rather than favorable outcomes alone.

Figure 9 shows that residual errors arise through several mechanisms rather than from a single class-specific failure. False-negative apical periodontitis appears in more than one example, consistent with the comparatively low recall of 0.6028 for this class. Decay is also detected at lower confidence in some cases, reflecting its limited radiographic contrast and 0.5303 mAP50-95. In densely restored regions, class confusion occurs among dental filling, root-canal filling, and porcelain-crown labels, while additional false-positive restorative predictions may appear near true crowns, fillings, or implants. The panel also contains an erroneous wisdom-tooth prediction and localization discrepancies in crowded posterior regions. These observations reinforce the class-wise quantitative results and show why strong localization of radiopaque restorative structures should not be generalized to subtle pathological findings.

### 4.8. IoU-Specific Localization Analysis

**Figure 10:**
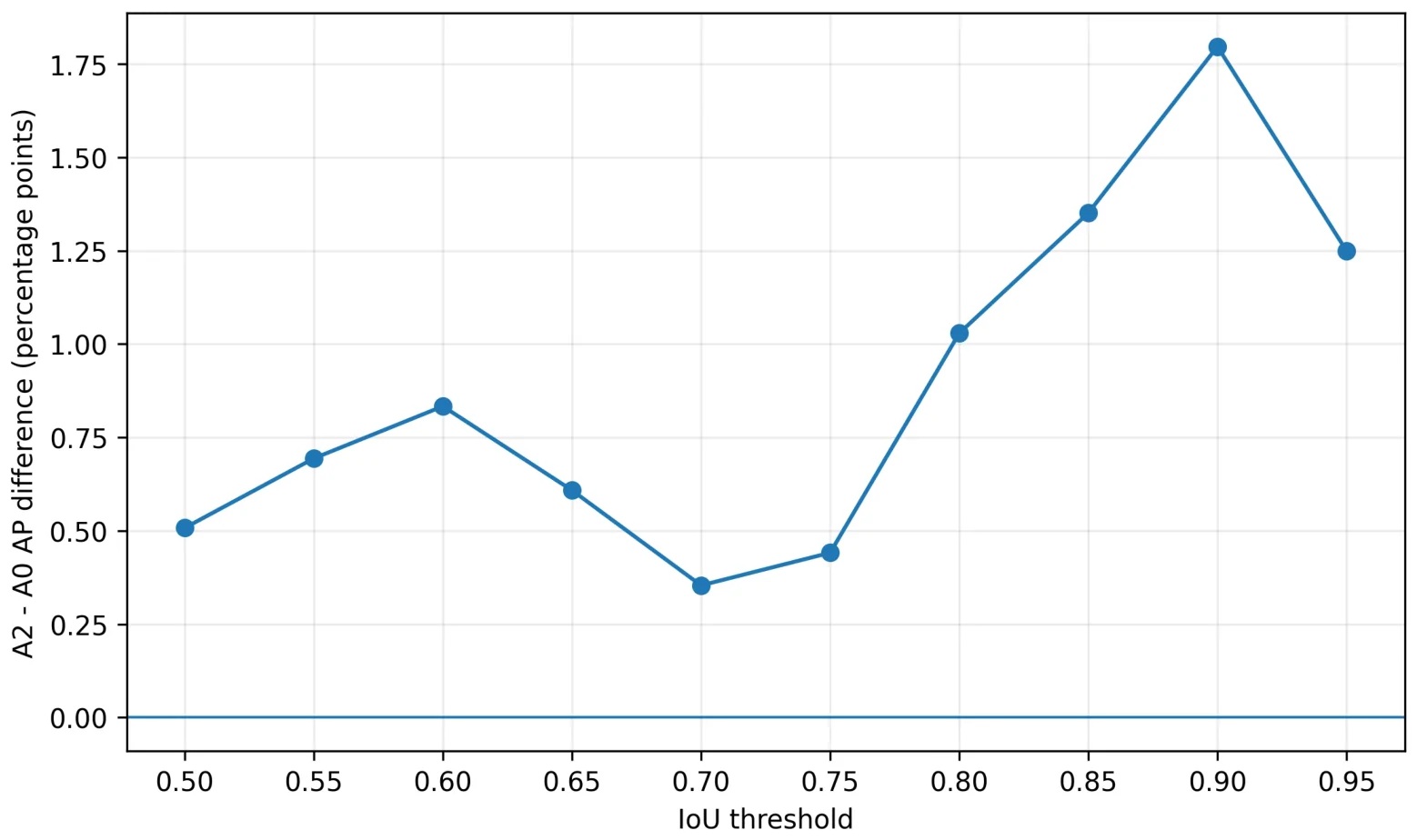
A2–A0 difference in mean AP across IoU thresholds from 0.50 to 0.95. Positive values indicate higher AP for A2 at the corresponding overlap requirement. The curve is a descriptive localization-sensitivity analysis of the reference checkpoints.

The A0–A2 difference remained positive at every evaluated IoU threshold from 0.50 to 0.95. The AP differences in percentage points at IoU thresholds 0.50, 0.55, 0.60, 0.65, 0.70, 0.75, 0.80, 0.85, 0.90, and 0.95 were +0.508, +0.694, +0.833, +0.608, +0.354, +0.441, +1.029, +1.351, +1.796, and +1.249, respectively. The trajectory is shown in Figure 10. The larger descriptive differences at 0.80–0.95 are compatible with improved strict-overlap behavior, but the pattern is not treated as a causal test of the boundary pathway in isolation.

Matched true-positive IoU distributions provided a complementary geometric summary. Using same-class greedy matching with confidence ≥ 0.25 and a minimum IoU of 0.50, A0 produced 17,606 matched detections with a mean/median IoU of 0.9024/0.9400 and an interquartile range of 0.8842–0.9679. A2 produced 17,734 matched detections with a mean/median IoU of 0.9046/0.9429 and an interquartile range of 0.8876–0.9690. Because the matched sets differ in size and are generated independently for each detector, these distributions are descriptive rather than a paired same-object hypothesis test.

## 5. Discussion

### 5.1. Architectural Interpretation and Strength of the Experimental Evidence

The improvement observed with SBRF-Det should be interpreted primarily in the context of the controlled staged ablation. The A0–A1–A2 comparison shows a progressive change in strict localization when the matched P4/16 stage is first replaced by RP-P4 and then by the complete SBRF formulation: mAP50-95 increased from 0.6934 to 0.7009 and 0.7029 in the matched single-run benchmark. The controlled multi-seed analysis preserved the same ordering across three distinct random seeds, with mean mAP50-95 values of 0.6909 ± 0.0012 for A0, 0.6957 ± 0.0005 for A1, and 0.6992 ± 0.0031 for A2. Paired radiograph-level resampling of the three-seed mean performance produced A2–A0 and A2–A1 differences of +0.822 and +0.344 percentage points, respectively. Together, these analyses show that the ordering is stable to the tested random-seed variation while confirming that the incremental A1-to-A2 improvement remains modest in absolute magnitude.

The component-removal analysis provides more direct evidence about the internal formulation while still requiring cautious interpretation. Removing the representative pathway, local structural pathway, or boundary-conditioned pathway reduced mAP50-95 by 0.440, 0.317, and 0.689 percentage points, respectively, relative to the complete A2 checkpoint. The fact that B2 retained nearly the same parameter count as A2 yet showed lower strict localization argues against parameter count alone as an explanation for the full-model result. At the same time, these B1–B3 experiments are single-run controlled removals; they support complementary contribution of the three pathways but do not justify a population-level ranking of component importance. Multi-run component ablation and interaction-focused factorial experiments would be required to quantify the uncertainty and interaction effects associated with each internal pathway.

The IoU-specific analysis provides a stricter check on the localization interpretation. A2 exceeded A0 at all ten IoU thresholds, with the largest descriptive differences at IoU 0.80–0.95, and matched true-positive mean/median IoU shifted from 0.9024/0.9400 to 0.9046/0.9429. This pattern is directionally consistent with improved box agreement under stricter overlap requirements. Nevertheless, the independently generated matched sets are not a one-to-one paired localization sample. The combined evidence therefore supports an association between the complete formulation and tighter localization without assigning the entire effect causally to the boundary gate.

The feature-scale and fusion sensitivity analyses further constrain the architectural interpretation. Holding M=25 across P3/8, P4/16, and P5/32 produced mAP50-95 values of 0.6912, 0.7027, and 0.6900, respectively, while the P4 gated-additive fusion variant reached 0.6916 compared with 0.7027 for the native asymmetric fusion. AP75 followed the same descriptive pattern. These results support the selected P4 placement and native fusion rule within the tested single-run protocol. They should not be interpreted as proof of universal optimality because moving the module changes the surrounding feature-stage context and these sensitivity comparisons were not repeated across multiple seeds.

Representative modeling also addresses a complementary property of panoramic radiographs: clinically relevant structures recur at distant locations across a wide field of view, and locally similar intensities may correspond to different findings depending on anatomical context. The Gather–Interact–Distribute formulation is conceptually related to Representative Attention [30]; however, RP-P4 was constructed as a detector-specific intermediate-stage representation pathway, and SBRF-Det extends it with a scale-dependent representative-count rule and boundary-conditioned local–global fusion. At the fixed 640 × 640 P4 setting, the implemented rule yields M=25, and the present experiments do not compare this rule against fixed representative budgets. The reported evidence therefore supports the integrated SBRF formulation and the component removals, but no isolated causal benefit is assigned to adaptive representative count. This distinction is important because the contribution of the present work lies in adapting compact global interaction to a heterogeneous multi-finding detection problem rather than simply inserting a generic attention block.

### 5.2. Clinical Interpretation and Pathological Findings

The class-specific results reveal a clinically important asymmetry that is partly obscured by aggregate detection metrics. Apical periodontitis remained the most difficult category, with recall of 0.6028 and mAP50-95 of 0.4526, while decay reached 0.5303 mAP50-95. In contrast, implant, porcelain crown, and ceramic bridge achieved substantially higher strict-localization performance. This difference is consistent with the radiographic characteristics summarized in Table 3: restorative and prosthetic structures are generally more radiopaque and structurally delimited, whereas early or subtle pathological changes may appear as low-contrast, spatially ambiguous radiolucencies.

The pathology-only aggregate reinforces this distinction. Across apical periodontitis and decay, A2 reached 0.7604 AP50, 0.5142 AP75, and 0.4916 mAP50-95, compared with 0.7458, 0.5082, and 0.4760 for A0. The positive changes show that the overall improvement was not confined to restorative labels, but the absolute pathology-only strict-localization level remains markedly below the full nine-class result. This is one reason the method is framed as computer-aided multi-finding localization rather than autonomous diagnosis.

The two-dentist qualitative assessment supports this interpretation. Examination of the complete 2688-image hold-out cohort identified successful multi-finding localization across all nine categories, but also documented clinically relevant false-positive, false-negative, class-confusion, and box-localization errors. False-negative apical periodontitis was visible in representative failure cases, and decay frequently showed lower confidence or less stable localization. In densely restored regions, confusion could occur among dental filling, root-canal filling, and porcelain-crown labels. These observations are important because an unflagged subtle pathological finding has a different clinical consequence from an incorrectly classified restorative structure. Accordingly, the strong performance observed for conspicuous treatment-related findings should not be generalized to disease detection, and SBRF-Det should not be used as an autonomous rule-out system on the basis of the present evidence.

A clinically realistic role for the detector would therefore be as a localization or second-read component that directs attention to candidate regions while leaving final interpretation to a dental professional. For screening or second-reader use, an operating point that prioritizes sensitivity for clinically important pathology may be preferable even if false-positive detections increase. The current benchmark does not determine that operating point, and the reported default threshold should not be interpreted as a clinically optimized decision threshold. Prospective threshold selection should be class specific and should explicitly consider the consequences of missed apical periodontitis and decay.

### 5.3. Statistical Sensitivity, Efficiency, and Reliability Trade-Offs

The multi-seed analysis provides direct evidence of robustness to random-seed variation across the three independently trained runs per configuration. Mean ± SD summarizes between-seed variability, whereas the paired radiograph-level bootstrap quantifies uncertainty in the corresponding three-seed mean differences on the released hold-out cohort. The two analyses therefore address complementary sources of variability. The bootstrap remains radiograph-level and cohort-specific and should not be interpreted as patient-clustered or multi-center uncertainty.

The class- and size-stratified secondary bootstrap shows that the aggregate change is not uniform. The medium-object interval excluded zero, whereas small- and large-object intervals did not. At class level, the 1000-resample intervals excluded zero for apical periodontitis, decay, wisdom tooth, dental filling, and root-canal filling, but not for missing tooth, implant, porcelain crown, or ceramic bridge. These results localize the checkpoint-level gain to a subset of classes and object scales rather than implying homogeneous improvement.

The operator trace also provides a direct execution-level explanation for the GFLOP/latency divergence. A2 increased bmm time from 19.006 to 35.525 ms across the profiled window and introduced 24.630 ms of einsum activity, while convolution time changed little. Accordingly, lower framework-reported GFLOPs should not be interpreted as evidence of lower wall-clock cost for the representative-routing formulation.

Finally, the non-identical-image analysis found no exact pHash match across development and hold-out partitions but did identify very high embedding similarity (maximum cosine 0.9998009). Excluding the most similar hold-out examinations did not remove the relative A2–A0 advantage in the secondary evaluator, but image similarity cannot identify subjects. The possibility of non-identical repeat examinations therefore remains a cohort-level limitation rather than a resolved leakage question.

Computational behavior also prevents the proposed model from being characterized as uniformly superior. In the dedicated final re-profiling, SBRF-Det used 26.950 M parameters and 93.06 GFLOPs compared with 26.396 M and 95.99 GFLOPs for A0. Despite the lower arithmetic count, pure inference latency increased from 5.946 to 8.851 ms/image and total validation-pipeline time from 6.618 to 9.540 ms/image, corresponding to pipeline throughputs of 151.1 and 104.8 images/s. Peak allocated GPU memory increased from 228 to 333 MB. This divergence illustrates that FLOPs, memory demand, and wall-clock execution are not interchangeable, particularly when representative routing, tensor reshaping, and feature-fusion operations introduce memory-access or kernel-launch overhead. Likewise, the calibration analysis does not identify SBRF-Det as the best-calibrated configuration; YOLO11l achieved lower ECE and Brier values in the reported comparison. The practical contribution of SBRF-Det is therefore concentrated in strict localization accuracy at moderate model size, not in universal dominance across speed, calibration, memory, and computational criteria.

### 5.4. Limitations and Future Work

The present evidence is subject to several limitations. All quantitative experiments were conducted on a single public panoramic-radiography benchmark. The released 2688-image hold-out test files did not provide stable patient identifiers that would permit reconstruction of repeated examinations or verification of subject-level separation from the development cohort. Exact-content and perceptual-hash screening found no byte-identical or exact perceptual duplicate across the development–hold-out boundary, but the embedding analysis identified highly similar radiographs, including a maximum cosine similarity of 0.9998009. Because visual similarity does not establish subject identity, patient-level independence and patient-clustered uncertainty could not be established. A directly compatible independent panoramic detection dataset with the same nine-class label space and comparable bounding-box definitions was also unavailable, so external generalizability was not tested. In addition, the placement and fusion sensitivity experiments were single-run analyses at 640×640, the adaptive representative-count rule was not isolated against fixed representative budgets, higher input resolutions were not evaluated, and no formal clinician reader study or clinically optimized class-specific operating thresholds were established. Performance for apical periodontitis and decay also remained substantially lower than for several restorative categories.

Future work should prioritize patient-linked multi-center validation using a harmonized nine-class annotation protocol, followed by repeated-seed component and interaction analyses. The scale-dependent representative rule should be compared directly with fixed representative budgets, and placement, fusion, and input-resolution sensitivity should be reassessed across repeated runs. Method development should place particular emphasis on apical periodontitis and decay, together with prospective class-specific calibration and threshold selection. Formal reader studies are also required to determine whether model assistance improves clinician sensitivity, interpretation time, and reliability before prospective workflow integration.

## 6. Conclusions

This study developed SBRF-Det for heterogeneous multi-finding localization in panoramic dental radiographs. The matched benchmark improved mAP50-95 from 0.6934 for A0 to 0.7029 for A2, and the three-seed analysis preserved the A0–A1–A2 ordering. Component-removal, fixed-M=25 placement, and fusion sensitivity analyses supported the tested P4/native-fusion configuration, although the overall gain was modest and accompanied by higher runtime. Performance remained weaker for apical periodontitis and decay than for restorative structures. SBRF-Det should therefore be regarded as a research localization framework that requires patient-linked multi-center validation, clinically defined operating thresholds, and formal reader evaluation before prospective clinical use. 

## Figures and Tables

**Table 1:** Architectural positioning of representative/global-context and feature-fusion strategies relative to RP-P4 and SBRF-Det.

Approach	Global/Context Mechanism	Local or Boundary Modeling	Scale-Adaptive Representative Allocation	Detector-Stage Role
Dense spatial self-attention	Direct token-to-token interaction over the spatial feature map	Not inherently tied to a task-specific local/boundary pathway	No bounded representative budget	Generic contextual modeling; computational cost grows with dense spatial interaction
Representative Attention [30]	Compact Gather–Interact–Distribute communication through representative features	Not formulated as the boundary-conditioned dental detection pathway used here	Not the scale rule introduced in SBRF-Det	Conceptual precursor for representative global communication in vision models
MMC-YOLO [20]	Progressive multi-scale and coordinate-guided contextual fusion	Edge-enhanced spatial fusion	No representative latent interaction	Dental multi-task context integration across scales
RP-P4 (ours)	Compact representative interaction inserted at the intermediate P4/16 stage	Retains the surrounding detector structure without the full SBRF boundary/local extension	No	Detector-specific representative feature pathway evaluated as A1
SBRF-Det (ours)	Representative interaction with a bounded, scale-dependent representative budget	Local depth-wise structural processing with boundary-conditioned modulation	Yes	Integrated P4/16 feature model for heterogeneous multi-finding localization

**Table 2:** Distribution of radiographs and annotated findings across the training, validation, and hold-out test sets.

Dataset Component/Finding	Training	Validation	Test	Total
Radiographs	8000	2000	2688	12,688
Annotated instances	52,500	12,960	18,639	84,099
Apical periodontitis	3614	899	1070	5583
Decay	7684	1935	1609	11,228
Wisdom tooth	17,675	4378	5272	27,325
Missing tooth	2764	668	660	4092
Dental filling	7543	1884	2212	11,639
Root canal filling	4937	1157	2340	8434
Implant	1759	443	2327	4529
Porcelain crown	3048	752	1652	5452
Ceramic bridge	3476	844	1497	5817

**Table 3:** Clinical definitions and characteristic panoramic radiographic appearances of the nine target findings.

Target Finding	Clinical Definition	Characteristic Panoramic Radiographic Appearance
Apical periodontitis	Inflammatory involvement of the apical periodontal tissues associated with infection and inflammation related to the root-canal system [31].	May begin with widening of the periodontal-ligament space and disruption of lamina-dura continuity; more established lesions can appear as radiolucent areas of variable size around the root apex [31].
Dental caries (decay)	A pathological process involving demineralization and progressive loss of dental hard tissues, including enamel, dentin, and cementum [32].	Usually appears more radiolucent than adjacent sound tooth structure in enamel and/or dentin; the shape and conspicuity vary with lesion location and depth [32].
Wisdom tooth	The third molar located at the most posterior aspect of the maxillary or mandibular dental arch [33].	Located distal to the second molar and may be erupted, partially erupted, or impacted; impacted third molars show variable angulation and intraosseous position [33].
Missing tooth	Absence of a tooth from the dental arch at a site where the tooth would otherwise be expected [34].	Characterized by absence of the expected dental structure and an edentulous region at the corresponding site [34].
Dental filling	Restoration of lost tooth structure using a restorative material [34].	Demonstrates material- and thickness-dependent radiopacity and is generally distinguishable from surrounding dental hard tissues by its radiodensity [35].
Root-canal filling	Obturation of the root-canal system with filling material after root-canal treatment [31].	Seen as radiopaque filling material extending along the root canal; length, density, and continuity can be assessed radiographically [31].
Dental implant	An endosseous prosthetic component placed within alveolar bone to support restoration of a missing tooth or teeth [34].	Typically appears as a sharply delineated radiopaque structure within alveolar bone; radiographic appearance depends on implant design and surrounding structures [34].
Porcelain crown	A fixed prosthetic restoration that surrounds the clinical crown of a tooth [34].	Envelops the coronal portion of the tooth and shows variable radiopacity according to the ceramic composition and the presence or type of underlying framework [34,35].
Ceramic bridge	A fixed multi-unit prosthetic restoration supported by teeth and/or implants to replace one or more missing teeth [34].	Appears as a connected multi-unit structure composed of retainers/crowns and pontic components, with radiopacity varying according to the restorative materials used [34,35].

**Table 4:** Hold-out test performance and original screening-profile complexity for all evaluated detectors. Parameter counts and GFLOPs in this table come from the fused-model summary used in the 22-model screening script; latency is the corresponding original batch-1 screening measurement.

Model	P	R	mAP50	mAP50-95	Params (M)	GFLOPs	Inference (ms/img)
SBRF-Det (A2)	0.8777	0.8979	0.9152	0.7029	26.90	92.22	8.56
RP-P4 (A1)	0.8774	0.8965	0.9133	0.7009	26.32	90.15	7.55
YOLOv10n	0.8551	0.8728	0.8926	0.6687	2.27	6.60	2.27
YOLOv10s	0.8625	0.8761	0.8973	0.6777	7.22	21.55	2.30
YOLOv10m	0.8643	0.8877	0.9032	0.6858	15.32	59.01	2.98
YOLOv10l	0.8765	0.8754	0.9046	0.6865	24.32	120.16	3.60
YOLOv10x	0.8716	0.8798	0.8972	0.6831	29.41	160.15	3.76
YOLO11n	0.8683	0.8750	0.9001	0.6737	2.58	6.38	2.37
YOLO11s	0.8782	0.8754	0.9034	0.6847	9.42	21.44	2.41
YOLO11m	0.8759	0.8936	0.9083	0.6924	20.04	67.80	2.95
YOLO11l	0.8722	0.8897	0.9048	0.6933	25.29	86.85	4.37
YOLO11x	0.8735	0.8838	0.9009	0.6885	56.84	194.81	5.64
YOLO12n	0.8649	0.8801	0.9002	0.6714	2.56	7.31	3.49
YOLO12s	0.8687	0.8861	0.9049	0.6875	9.23	23.19	3.56
YOLO12m	0.8818	0.8784	0.9076	0.6915	20.11	70.42	3.83
YOLO12l	0.8785	0.8871	0.9076	0.6934	26.35	95.13	6.07
YOLO12x	0.8792	0.8817	0.9082	0.6909	59.05	208.39	6.58
YOLO26n	0.8519	0.8724	0.8915	0.6652	2.38	5.32	2.74
YOLO26s	0.8607	0.8678	0.8918	0.6717	9.47	20.78	2.86
YOLO26m	0.8664	0.8748	0.8974	0.6840	20.36	68.12	3.11
YOLO26l	0.8589	0.8876	0.8974	0.6840	24.75	86.49	4.29
YOLO26x	0.8576	0.8805	0.8951	0.6829	55.64	193.96	5.56

**Table 5:** Progressive ablation of the proposed P4 feature-modeling strategy. Complexity columns retain the fused-model screening convention used in Table 4.

Variant	P	R	mAP50	mAP50-95	Δ mAP50-95 vs. A0	Params (M)	GFLOPs	Latency (ms/img)
A0: baseline	0.8785	0.8871	0.9076	0.6934	–	26.35	95.13	6.07
A1: RP-P4	0.8774	0.8965	0.9133	0.7009	+0.75 pp	26.32	90.15	7.55
A2: SBRF-Det	0.8777	0.8979	0.9152	0.7029	+0.95 pp	26.90	92.22	8.56

**Table 6:** Controlled internal-pathway and architecture-sensitivity analyses of SBRF. (**A**) removes one internal pathway from the complete A2 formulation. (**B**) evaluates feature-scale placement at fixed M=25 and a gated additive fusion alternative at P4. All entries are single-run comparisons under the predefined 300-epoch, seed-0, 640×640, batch-4 protocol; the native P4 row uses the reference A2 checkpoint.

**(A) Internal-pathway removal**						
**Variant**	**P**	**R**	**mAP50**	**mAP50-95**	**Δ vs. A2, pp**	**Params (M)**
A2: full SBRF	0.8774	0.8982	0.9153	0.7028	–	26.95
B1: no representative pathway	0.8758	0.8952	0.9123	0.6984	−0.440	25.80
B2: no local pathway	0.8744	0.8924	0.9114	0.6996	−0.317	26.93
B3: no boundary pathway	0.8712	0.8941	0.9093	0.6959	−0.689	26.40
**(B) Feature-scale and fusion sensitivity**						
**Variant**	M	**mAP50**	**AP75**	**mAP50-95**	**Δ vs. native P4, pp**	
P3/8, native fusion	25	0.9094	0.7255	0.6912	−1.147	
P4/16, native fusion	25	0.9153	0.7297	0.7027	–	
P5/32, native fusion	25	0.9128	0.7237	0.6900	−1.270	
P4/16, gated additive fusion	25	0.9121	0.7240	0.6916	−1.111	

**Table 7:** Final controlled batch-1 computational profile of the focal baseline, proposed, and component-removal variants at 640 × 640 input resolution using the loaded-checkpoint profiling convention. Pipeline time is the sum of preprocessing, inference, and postprocessing reported by the validation framework. FPS is derived from pipeline time.

Variant	Params (M)	GFLOPs	Inference (ms/img)	Pipeline (ms/img)	Pipeline FPS	Peak GPU (MB)
A0: baseline	26.396	95.99	5.946	6.618	151.1	228
A1: RP-P4	26.368	90.99	7.842	8.526	117.3	296
A2: full SBRF	26.950	93.06	8.851	9.540	104.8	333
B1: no representative pathway	25.802	88.81	5.908	6.590	151.8	328
B2: no local pathway	26.932	93.00	8.727	9.415	106.2	297
B3: no boundary pathway	26.401	91.29	8.198	8.885	112.5	332

**Table 8:** Class-specific SBRF-Det performance and secondary A0–A2 uncertainty analyses on the same 2688-radiograph hold-out cohort. (**A**) reports class-specific precision, recall, F1-score, mAP50, and mAP50-95 of SBRF-Det. (**B**) reports the bootstrap mean A2–A0 mAP50-95 difference and percentile 95% interval from 1000 paired radiograph-level resamples. (**C**) reports COCO-style size-stratified AP from the reference A0 and A2 checkpoints with paired intervals for the difference.

**(A) SBRF-Det class-specific performance**							
**Class**	**Images**	**Instances**	**P**	**R**	**F1**	**mAP50**	**mAP50-95**
Apical Periodontitis	683	1070	0.8188	0.6028	0.6944	0.7402	0.4526
Decay	953	1609	0.7282	0.8091	0.7665	0.7801	0.5303
Wisdom Tooth	1970	5272	0.9562	0.9885	0.9721	0.9884	0.8172
Missing Tooth	466	660	0.8072	0.9409	0.8690	0.9483	0.6050
Dental Filling	977	2212	0.8574	0.8373	0.8472	0.8930	0.6750
Root Canal Filling	1184	2340	0.8265	0.9406	0.8799	0.9217	0.6451
Implant	970	2327	0.9897	0.9953	0.9925	0.9894	0.8401
Porcelain Crown	960	1652	0.9660	0.9805	0.9732	0.9889	0.8842
Ceramic Bridge	861	1497	0.9498	0.9863	0.9677	0.9872	0.8766
**(B) Paired class-specific A2–A0 mAP50-95 differences**							
**Class**		**Bootstrap mean Δ, pp**			**95% CI, pp**		
Apical Periodontitis		+1.540			[0.563, 2.516]		
Decay		+1.287			[0.347, 2.224]		
Wisdom Tooth		+0.727			[0.371, 1.090]		
Missing Tooth		+1.133			[−0.102, 2.434]		
Dental Filling		+1.692			[1.120, 2.333]		
Root Canal Filling		+1.275			[0.612, 1.943]		
Implant		+0.211			[−0.275, 0.663]		
Porcelain Crown		+0.183			[−0.393, 0.764]		
Ceramic Bridge		+0.061			[−0.588, 0.759]		
**(C) Size-stratified checkpoint-level AP**							
**Object stratum**		**A0 AP**	**A2 AP**	**Observed Δ, pp**	**95% CI for Δ, pp**		
Small		0.1855	0.1864	+0.091	[−0.646, 0.917]		
Medium		0.6225	0.6309	+0.838	[0.261, 1.396]		
Large		0.6963	0.6985	+0.224	[−0.511, 0.980]		

**Table 9:** Multi-seed robustness summary and paired 10,000-iteration radiograph-level bootstrap for A0, A1, and A2.

Model/Comparison	mAP50, Mean ± SD	mAP50-95, Mean ± SD	Δ mAP50-95 (95% CI), pp
A0: baseline	0.9055 ± 0.0025	0.6909 ± 0.0012	–
A1: RP-P4	0.9100 ± 0.0013	0.6957 ± 0.0005	+0.478 vs. A0 [0.301, 0.628]
A2: SBRF-Det	0.9128 ± 0.0023	0.6992 ± 0.0031	+0.822 vs. A0 [0.663, 0.998]
A2 vs. A1	–	–	+0.344 [0.215, 0.514]

## Data Availability

The radiographs and annotations analyzed in this study are available through the public OralXrays-9 dataset described by Chen et al. [19], subject to the terms of the source release. Derived split definitions, evaluation summaries, and other non-identifiable study outputs can be provided by the corresponding authors upon reasonable request, subject to the original dataset license and distribution conditions. The experiments were implemented using Ultralytics 8.4.121 with the proposed RP-P4 and SBRF modules and the model configurations described in this manuscript. Source-code components required to reproduce the architecture, together with model configuration files, derived split manifests, evaluation scripts, operator-level profiler CSV/trace outputs, and the secondary bootstrap and similarity-analysis summaries, are available from the corresponding authors upon reasonable request, subject to the OralXrays-9 distribution terms and third-party software licenses.
